# Time-resolved, single-cell analysis of induced and programmed cell death via non-invasive propidium iodide and counterstain perfusion

**DOI:** 10.1038/srep32104

**Published:** 2016-09-01

**Authors:** Christina E. M. Krämer, Wolfgang Wiechert, Dietrich Kohlheyer

**Affiliations:** 1 IBG-1: Biotechnology, Forschungszentrum Jülich GmbH, Jülich, Germany

## Abstract

Conventional propidium iodide (PI) staining requires the execution of multiple steps prior to analysis, potentially affecting assay results as well as cell vitality. In this study, this multistep analysis method has been transformed into a single-step, non-toxic, real-time method via live-cell imaging during perfusion with 0.1 μM PI inside a microfluidic cultivation device. Dynamic PI staining was an effective live/dead analytical tool and demonstrated consistent results for single-cell death initiated by direct or indirect triggers. Application of this method for the first time revealed the apparent antibiotic tolerance of wild-type *Corynebacterium glutamicum* cells, as indicated by the conversion of violet fluorogenic calcein acetoxymethyl ester (CvAM). Additional implementation of this method provided insight into the induced cell lysis of *Escherichia coli* cells expressing a lytic toxin-antitoxin module, providing evidence for non-lytic cell death and cell resistance to toxin production. Finally, our dynamic PI staining method distinguished necrotic-like and apoptotic-like cell death phenotypes in *Saccharomyces cerevisiae* among predisposed descendants of nutrient-deprived ancestor cells using PO-PRO-1 or green fluorogenic calcein acetoxymethyl ester (CgAM) as counterstains. The combination of single-cell cultivation, fluorescent time-lapse imaging, and PI perfusion facilitates spatiotemporally resolved observations that deliver new insights into the dynamics of cellular behaviour.

“Alive or dead?”, “How dead is dead?” or “How red is dead?” are pivotal questions posed during cellular live/dead determination, particularly when *in vivo* staining is performed with propidium iodide (PI). Although PI is a common cell death indicator, a gold standard protocol for its use does not exist, and inconsistent staining results and pitfalls have been reported in the literature^1^,^2^,^3^,^4^,^5^,^6^.

PI is a versatile indicator dye for dead cells that acts by intercalating with cellular DNA and emitting red fluorescence. Vital staining with PI is dependent on the impermeability of an intact cell membrane to this molecule. Live/dead staining with PI is commonly implemented to evaluate the viability of bacteria sampled from food products, clinical samples, and environmental or fermentation processes and to characterize vitality in eukaryotic cells^1^,^7^,^8^. This staining procedure has been employed for bacteria^2^,^3^, biofilms^9^, yeasts^1^, and a variety of mammalian cells^10^. However, the toxicities of fluorescence indicators or certain concentrations are rarely considered.

Microscopic imaging approaches employing microfluidic devices containing cells prestained with PI and cell-wall permeant SYTO 9 have been reported for the live/dead quantification of bacterial cells^11^,^12^,^13^, sperm cells^14^, and yeast^15^ and are, in principle, comparable to studies using fluorescence activated cell sorting (FACS). Conventional staining protocols using PI concentrations higher than 1 μM intended for sorting^4^,^14^, confirmation of cell lysis^16^, or cellular analytics^17^,^18^,^19^,^20^ have been described for prokaryotes and eukaryotes. PI staining is generally performed as an endpoint measurement, frequently after cell fixation^17^,^19^,^21^.

PI is often, but not exclusively, used in combination with SYTO 9 as a counterstain^2^,^4^,^5^,^22^. PI is also combined with other cell-permeable DNA dyes, such as other SYTO dyes (*e.g.*, SYTO 15 and SYTO 13)^17^,^23^, acridine orange^19^,^24^, SYBR green^6^ and SYTOX dyes (*e.g.*, SYTOX Red and SYTOX Green)^20^,^25^ to facilitate total cell staining. Alternatively, prokaryotic viability can be assessed via bacterial GFP expression prior to PI staining in lieu of a total cell stain^2^,^9^,^17^. Furthermore, PI is used in combination with the monomeric cyanine dyes PO-PRO-1 and YO-PRO-1^20^, the green fluorogenic esterase substrate calcein-acetoxymethyl ester (CgAM)^8^,^18^,^21^,^26^, a fluorescent caspase inhibitor^27^, annexin V^28^, or specific overall stains (*e.g.*, ConA-Alexa Fluor 488^29^, FITC-dextran^10^, and Cell Tracker Green^21^). In addition, cancer cells have been continuously perfused with PI to demonstrate the efficiency of live cell trapping in a media stream or to indicate the need for cytotoxicity testing with microfluidic devices^20^,^30^.

We present a dynamic and non-invasive cell viability staining method employing a low PI concentration in combination with non-toxic counterstains, provided continuously to bacteria or yeast in a microfluidic growth chamber array. Staining experiments utilized a microfluidic PDMS-glass device designed for single-cell studies of *Corynebacterium glutamicum* and *Escherichia coli*^31^,^32^,^33^. This cultivation approach, in combination with time-lapse fluorescence microscopy, confers spatiotemporal insight into the phenotypic variations and dynamics of cellular death at the single-cell level. In particular, the dynamic heterogeneity of cell phenotypes, such as differences in tolerance, resistance, or epigenetic predisposition among isogenic cultures, can be analysed during well-controlled perturbation studies. Exogenous and endogenous cell death triggers were applied to test our ability to perform temporally resolved cell death analysis of *C. glutamicum*, *E. coli* and *Saccharomyces cerevisiae*, emphasizing the generic analytical approach. PI invasion of fungal and bacterial cells in combination with non-toxic counterstains (violet fluorogenic calcein acetoxymethyl ester (CvAM), CgAM, and PO-PRO-1) is indicative of both sudden and prolonged cell death. Thus, the immediately apparent fluorescence responses of this novel and dynamic staining approach facilitate the elucidation of survival strategies in small cell populations, such as antibiotic tolerance, temporal resistance, resuscitation after membrane potential loss, or the formation of membrane permeability transition pores (MPTPs). The technical approach presented here permits the temporally resolved observation of phenotypic variations at the single-cell level. The spatial resolution of intracellular fluorescence distribution enables the visualization of specific details regarding cell death, such as the partial death of cell poles after antibiotic contact.

## Results

### PI concentration optimization

In the present study, the conventional viability assay employing PI and SYTO 9, a multi-step method^22^, was transformed into a single-cell, one-step method resolved in real time with microbial cultivation occurring inside a microfluidic device. This microfluidic perfusion system ensures the continuous presence of extracellular PI at a specific concentration for all cells during experiments. For a more detailed description of the constant microfluidic perfusion environment, the interested reader is referred to the Supplementary Information and Fig. S1.

**Figure 1:**
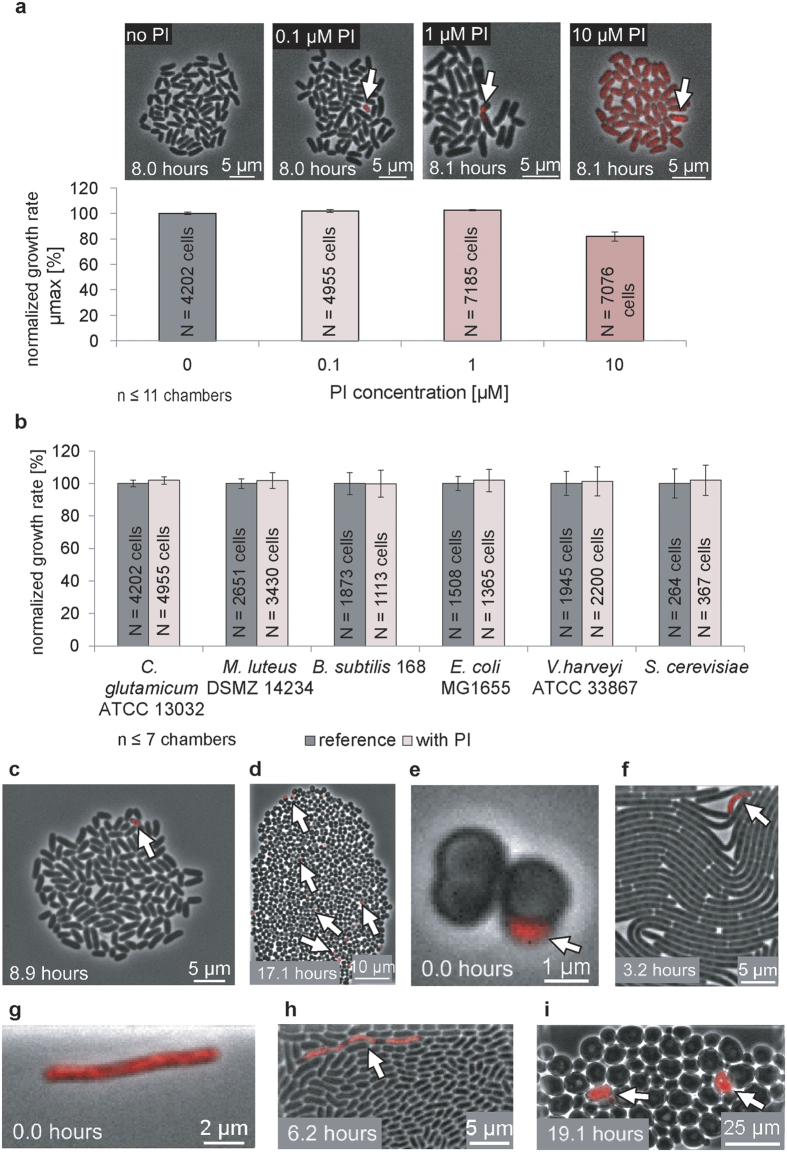
Determination of optimal propidium iodide concentration. (**a**) The model organism *Corynebacterium glutamicum* was stained continuously with 0.1 μM PI, 1 μM PI, and 10 μM PI, and bacterial growth was normalized to the growth rate without PI addition. Total cell numbers are indicated with N. PI^+^ dead cells are marked by white arrows. (**b**) A PI concentration of 0.1 μM was used with Gram-positive bacteria (*C. glutamicum* ATCC 13032, *Micrococcus luteus* DSMZ 14234 and *Bacillus subtilis* 168), Gram-negative bacteria (*Escherichia coli* MG1655 and *Vibrio harveyi* ATCC 33867), and a small eukaryote (*Saccharomyces cerevisiae*). The growth rates of all microorganisms cultivated with 0.1 μM PI were normalized and compared to reference colonies grown without PI. Total cell numbers are given by N. **(c)** *C. glutamicum* ATCC 13032 colony with a single PI^+^ cell. (**d**) *M. luteus* DSMZ 14234 colony in the late exponential phase with distributed PI^+^ cocci. (**e**) *M. luteus* DSMZ 14234 tetrad with the early appearance of a PI^+^ cell. (**f**) Densely grown *B. subtilis* 168 cell colony with the late appearance of a PI^+^ cell. **(g)** Early appearance of a PI^+^ *E. coli* MG1655 cell. **(h)** Segmented *V. harveyi* ATCC 33867 PI^+^ phenotype in a cell-packed region. **(i)** Dense *S. cerevisiae* colony with PI^+^ yeast cells.

The Gram-positive bacterium *C. glutamicum* was cultivated with minimal medium (CGXII + 4% glucose (w/v) without PI) and used as the reference for three different PI concentrations (0.1 μM, 1 μM, and 10 μM). *C. glutamicum* growth was impaired by 10 μM PI. PI permeated and slightly stained intact cells, but these bacteria continued to grow, although at a reduced rate. Bacterial growth was unimpaired by concentrations of 0.1 or 1 μM PI (Fig. 1a). However, positively stained cells (PI^+^) were observed at frequencies of <0.01% for all three PI concentrations due to spontaneous single cell death.

Based on these data, a PI concentration of 0.1 μM was employed for our microfluidic analyses and validated by the addition of phenol during *C. glutamicum* cultivation (see Supplementary Information Fig. S2). Furthermore, 0.1 μM PI was found to be non-toxic and universally applicable, as revealed by testing a wide range of microorganisms cultivated in different complex media, including *Micrococcus luteus* (1.78% PI^+^)*, Bacillus subtilis* (0.09% PI^+^)*, E. coli* (<0.01% PI^+^)*, Vibrio harveyi* (<0.01% PI^+^) and the yeast *S. cerevisiae* (2.72% PI^+^) (Fig. 1b). A positive control involving additional PO-PRO-1 staining during cyanide intoxication confirmed PI as rapid and precise cell death detection system during cultivation (see Fig. S4, Supplementary Information).

The tested microbes were selected for their diverse cell-wall structures and taxonomic variations. Independent of cell-wall structure, membrane disintegration was instantaneously observable during cultivation. Compared to reference cultures, microorganismal growth was not influenced by the addition of 0.1 μM PI. However, a negligible fraction of cells was PI^+^ directly following inoculation and at the end of cultivation when the cultivation chambers were was nearly or completely filled with cells (Fig. 1c–i). Classification criteria based on fluorescence signals are described in detail in the Material and Methods section.

An *M. luteus* tetrad with a dead coccus and a dead *E. coli* cell obtained pre-culture directly after seeding are shown in Fig. 1e,g, respectively. PI^+^ cells randomly distributed in culture at the end of cultivation are shown for *C. glutamicum* (Fig. 1c), *M. luteus* (Fig. 1d), *B. subtilis* (Fig. 1f), *V. harveyi* (Fig. 1h), and *S. cerevisiae* (Fig. 1i).

### Prokaryotic cell death and apparent antibiotic tolerance following the addition of antibiotics

On-line viability monitoring with PI was performed for the non-pathogenic organism *C. glutamicum*, which is related to various human pathogens (*e.g., Mycobacterium tuberculosis* and *Corynebacterium diphtheriae*)^34^, in the continuous presence of environmental antibiotic concentrations. Dynamic PI staining was validated by the addition of CvAM as a non-invasive counterstain, which is converted intracellularly to violet fluorescent calcein (CALv), as described recently^32^. Viable cells exhibited moderate CALv fluorescence, whereas apparent tolerant cells with reduced metabolism were intensely fluorescent^32^. Dual staining had no impact on growth (Supplementary Information, Fig. S4), and additional excitation during multiplexed fluorescent time-lapse imaging was tested and shown to be non-phototoxic (Fig. S5).

*C. glutamicum* was cultivated during the perfusion of constant concentrations of ethambutol (EMB), ampicillin (AMP), kanamycin (KAN), streptomycin (STR), and chloramphenicol (CHL). These antimicrobials are categorized as bactericidal inhibitors of cell-wall synthesis (EMB and AMP), bactericidal aminoglycoside antibiotics causing mRNA misreading and inhibit protein biosynthesis (KAN, STR), and a bacteriostatic protein synthesis inhibitor (CHL)^35^. Heterogeneous fluorescence distribution attributable to concentration gradients was not observed (data not shown).

The cells continued asymmetric division for more than 9 hours with 25 mg/mL EMB, whereas division halted after the second filial generation with 25 μg/mL AMP and after the first cell division with 25 μg/mL KAN and 50 μg/mL STR. Cell division ceased after initial antibiotic contact with 50 μg/mL KAN and 50 μg/mL CHL (Video S1).

**Figure 2:**
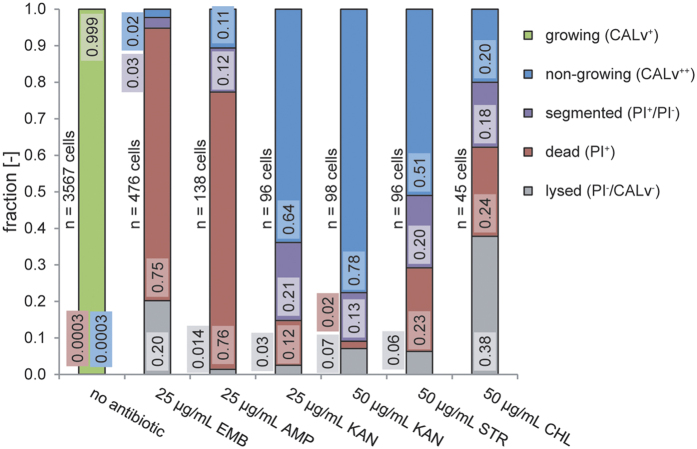
Fractions of different cell states following continuous antibiotic treatment of *C. glutamicum* ATCC 13032 cells at 25 μg/mL for 12 h and at 50 μg/mL for 16.6 h. Dead cells are identified by a significant increase in PI fluorescence (PI^+^/CALv^−^), antibiotic-tolerant cells are identified by the conversion of CvAM to CALv and its retention (PI^−^/CALv^+^), lysed cells are non-fluorescent and pale in phase contrast images (PI^−^/CALv^−^), segmented cells are bipolar with a dead pole and a tolerant pole (PI^+^/CALv^−^/PI^−^/CALv^+^). Growing cells were defined as non-inhibited with respect to cell elongation for *C. glutamicum*, whose cells typically undergo snapping cell division. The bactericidal antibiotics EMB and AMP are inhibitors of cell wall synthesis. The bacteriostatic mRNA inhibitor KAN was tested at 25 μg/mL and 50 μg/mL. STR and the bacteriostatic CHL inhibit protein biosynthesis.

**Figure 3:**
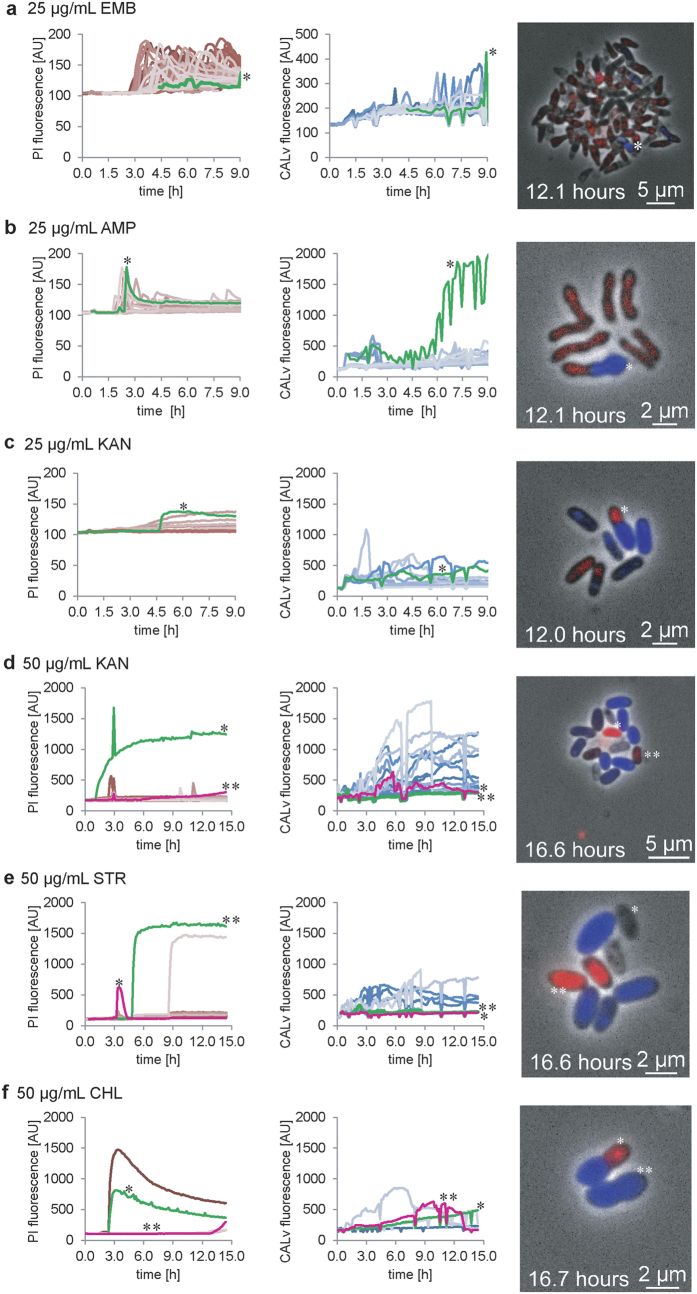
Antibiotic-induced cell death and antibiotic tolerance of wild-type *C. glutamicum* ATCC 13032. Mean single-cell fluorescence traces are shown for single cells from one representative growth chamber stained continuously with the cell death indicator PI (right) and the metabolic activity indicator CALv (middle). The micrographs (right) show representative cells from the final time-lapse images. Dead (PI^+^/CALv^−^), lysed (PI^−^/CALv^−^), antibiotic-tolerant (PI^−^/CALv^+^), or segmented cells with a dead cell pole and a surviving cell pole (PI^+^/CALv^−^/PI^−^/CALv^+^) can be distinguished. Cells were continuously treated with **(a)** 25 μg/mL EMB (a deformed cell that retained cell wall integrity is marked with *), **(b)** 25 μg/mL AMP (a deformed cell that partially retained CALv fluorescence is marked with *), **(c)** 25 μg/mL KAN (a segmented cell with a cell pole that is PI^+^ and a PI^−^ cell pole with CALv fluorescence is marked with *), **(d**) 50 μg/mL KAN (heterogeneous PI^+^ cells with bright PI fluorescence (*) and pale PI fluorescence (**) are marked), **(e**) 50 μg/mL STR (dead, lysed cells with rapid PI fluorescence loss (*) and dead cells with constant PI fluorescence (**) are compared), **(f)** and 50 μg/mL CHL (segmented cells with halted growth and independently stained cell poles; two cells are marked, one with a PI^+^ pole and a blue fluorescent PI^−^ pole (*) and another with two cell poles exhibiting CALv fluorescence (**)).

Cell growth was impaired via different mechanisms, resulting in phenotypic variation, as shown in Figs 2 and 3 and described in the Supplementary Information. The status of antibiotic-treated cells was altered, as indicated by intracellular fluorescence. In contrast to untreated cells (Fig. 2, no antibiotics), the addition of antibiotics resulted in formation of a subpopulation of highly CALv-fluorescent non-growing cells (PI^−^/CALv^++^) that were considered apparent antibiotic tolerant as well as subpopulations of dead PI^+^ cells (PI^+^/CALv^−^) and non-fluorescent cells (PI^−^/CALv^−^) that lost their intracellular content following lysis. Fractions of dead and lysed cells differed according to the antibiotic applied (cell wall synthesis or translational processes); see Fig. 2.

Mean colony fluorescence with a high standard deviation confirmed changes in individual fluorescence profiles based on antibiotic treatment (see Fig. 3, Fig. S7 and Supplementary Information). Bacteria with injured cell walls lost intracellular CALv fluorescence while PI intruded and underwent DNA intercalation (Fig. S8). The mean single-cell PI fluorescence equilibrium differed according to the antibiotic. Cell wall-impairing antibiotics (EMB and AMP) resulted, by far, in the lowest mean single-cell PI fluorescence values (Fig. 3 and Fig. S7). Heterogeneous PI^+^ cells were observed with 50 μg/mL KAN (Fig. 3d, cells marked with *).

Bacterial growth arrest was not a specific indicator of cell death, as several cells remained unstained by PI (PI^−^) even after growth halted. Residual CALv fluorescence revealed bacterial survival among cells subjected to treatment with all six antibiotics, even after 16 hours (apparent antibiotic tolerance). Thus, PI fluorescence indicates bacteria that are permeabilized by an antibiotic. Cell membrane disintegration and a concurrent increase in PI fluorescence differed among single cells by antibiotic (Fig. 3).

PI^+^ cells exhibited increased maximum mean single-cell fluorescence values of 1000–1500 AU. PI^ + ^cells continuously cultivated at 25 μg/mL AMP (Fig. 3b, *), 50 μg/mL STR (Fig. 3e, *) or 50 μg/mL CHL (Fig. 3f, *) showed subtle decreases in mean single-cell fluorescence over time. Although the fluorescence profiles of these cells resembled a Bateman function, fluorescence loss was more rapid than that induced by photo bleaching, which accounted for 1% to 3% of the total mean single-cell PI fluorescence across all imaging frames (Fig. S4).

Lysed cells that retained their cell shape as visible ghost cells demonstrated rapid reductions in intracellular PI and CALv fluorescence due to DNA loss and cell membrane destruction (Fig. 3e, cell marked with *, Fig. S8). In contrast, the PI fluorescence values of dead bacteria rapidly reached high and stable plateaus (Fig. 3e, cell marked with **). Reduced PI fluorescence in these cells correlated with decreased DAPI signals as determined by additional end-point staining of total DNA (Fig. S8), indicating possible DNA decay or fractional DNA loss.

Furthermore, the apparent fractional tolerance of segmented cells exhibited two different viable states. In addition to fully living cells, segmented cells with dead cell poles that were PI^ + ^and cell poles that retained cell wall integrity were observed in the presence of 50 μg/mL CHL (Fig. 3c,f, cells marked with * and **, respectively).

However, a fraction of the bacteria remained PI^−^ while demonstrating remarkably increased CALv fluorescence (CALv^ +  + ^). These cells did not stain red or lyse and tolerated continuous antibiotic treatment in a non-growing but metabolically active state during the observed time frame (Fig. 3a,b, cells and single-cell fluorescence traces marked with *).

### Programmed cell death (PCD) of *E. coli*

**Figure 4:**
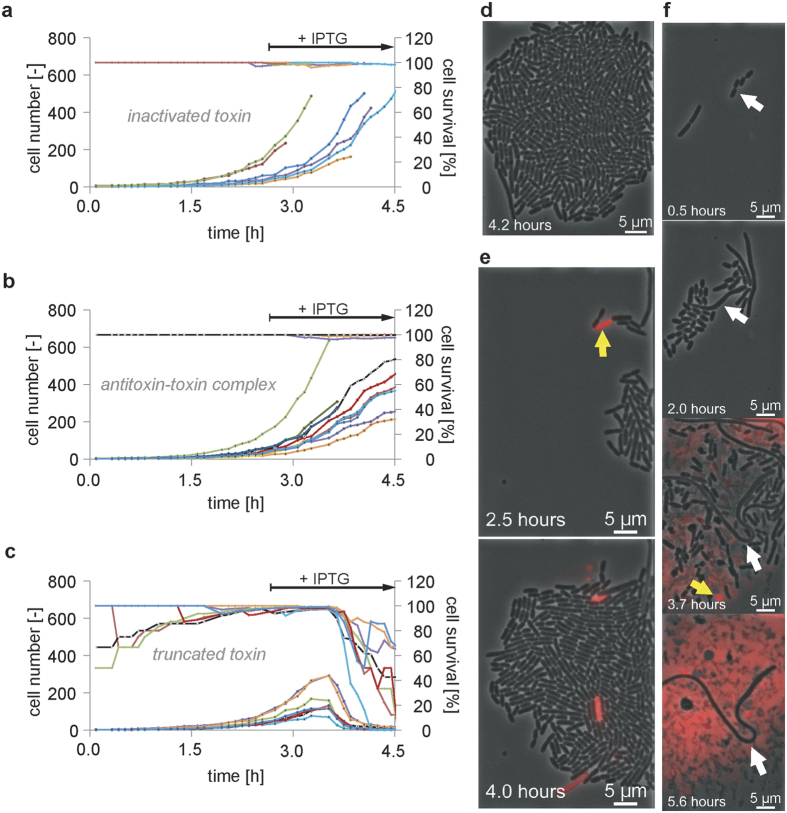
PI to determine the bacterial survival rate following toxin-antitoxin module expression in *E. coli* BL21CodonPlus(DE3)-RIL. Strains harbouring the plasmids pET28b(*pezTΔ*C_242_(*D66T*)), pET28b(*pezA/pezT*), or pET28b(*pezTΔ*C_242_) produced **(a)** an inactivated toxin, **(b)** the antitoxin-toxin complex or **(c)** the C-terminal truncated toxin. Micrographs showing an *E. coli* colony with **(d)** inactivated toxin expression, **(e)** a colony of cells before and after expression of the antitoxin-toxin module, and **(f)** a colony expressing the truncated toxin, which caused cell lysis, are presented. Cells stained prior to IPTG induction (2.6 h) or prior to lysis are indicated with a yellow arrow. A white arrow indicates a cell that resisted toxin expression but did not resuscitate following a backshift to growth medium.

Toxin-antitoxin modules are present in a wide range of bacteria. Their functions and triggers are currently under intensive investigation to determine their potential antimicrobial use. In the present study, we performed dynamic PI staining and undertook the time-resolved observation of a pneumococcal zeta toxin (PezT) described by Mutschler *et al*.^36^. *E. coli* BL21CodonPlus(DE3)-RIL bearing pET28b(*pezTΔ*C_242_) (Fig. 4a), pET28b(*pezA/pezT*) (Fig. 4b), or pET28b(*pezTΔ*C_242_(*D66T*)) (Fig. 4c) expressed an inactivated toxin, the C-terminal truncated toxin or the antitoxin-toxin complex, respectively, after induction with 100 μM IPTG after 2.6 h of cultivation (Video S2).

Cells underwent lysis after expressing the truncated toxin, which impairs cell wall synthesis. Extracellular DNA was stained immediately and exhibited red fluorescence. Non-lysed PI^ + ^cells were observed when toxin-producing mutants were cultivated but were rarely observed (at a frequency of 1.4%) during cultivation of the strain expressing the antitoxin-toxin complex (Fig. 4e,f, yellow arrow, and Video S2). Thus, cell death occurred independent of induced toxin production. Rare cells containing the truncated toxin-bearing plasmid remained in a non-replicating PI^−^ state at a frequency of 1.5% (Fig. 4f). These resistant cells did not die and were not lysed during 5.6 h of IPTG treatment (Fig. 4f, white arrow). Recovery was not achieved by reverting to an LB medium without the inducer more than 4 h after IPTG induction. Resuscitation was attempted until all resistant cells became PI^ + ^(data not shown).

Continuous PI fluorescent time-lapse imaging enabled us to distinguish between toxin-induced lytic cell death, incidental non-lytic cell death and toxin resistance. Prior to the induction of toxin expression, single cells exhibited PI fluorescence and were overgrown by non-fluorescent bacteria until toxin production began (Fig. 4c). Therefore, the percentage of live cells increased to almost 100% and then decreased subsequent to toxin production.

### Temporally resolved PCD in yeast

Baker’s yeast is a simple model organism used to study apoptotic phenotypes and lethal cell differentiation. Given that starvation induces cell death and PCD in *S. cerevisiae*^37^, we analysed yeast cells during microfluidic cultivation with a fresh supply of YPD medium combined with dynamic dual staining using PI/CgAM or PI/PO-PRO-1 after pre-cultivation in a shaking flask under famine conditions. Famine conditions were initiated by (i) medium replacement with 0.9% NaCl (w/v) (starvation conditions) and (ii) prolonged pre-cultivation in YPD (nutrient limitation conditions).

In contrast to the rapid necrosis that occurred among yeast cells in-between the 30-min imaging period, which was also observed in reference experiments, apoptotic phenotypes exhibited death rates that were relatively prolonged, as described below. Yeast PCD involves a complex functional network^38^, and interactions between PCD and cell ageing, mating, and autophagy pathways, as well as the epigenetics of PCD, have been recently reviewed^39^,^40^,^41^,^42^,^43^. In contrast to apoptotic PCD, which is characterized by genetic regulation and energy dependence, necrosis occurs in an uncontrolled manner after the swelling of cells or organelles, sudden loss of plasma membrane integrity, or the occurrence of cellular dysfunction^44^.

Nutrient stress during pre-cultivation promotes chronological ageing, triggering PCD^40^. We observed a variety of single-cell death phenotypes in yeast that were temporally resolved by fluorescent time-lapse imaging with dual staining using either PI/PO-PRO-1 or PI/CgAM (Supplementary Information, Fig. S8, S9 and Videos S3,S4,S5,S6).

Given that PO-PRO-1 stains dsDNA via intercalation comparable to PI, time-resolved, single-cell, dual-fluorescence imaging permitted us to distinguish necrosis (a sudden change from PO-PRO-1^−^/PI^−^ to PO-PRO-1^ + ^/PI^ + ^) and apoptosis (PO-PRO-1^ + ^/PI^−^ to PO-PRO-1^ + ^/PI^ + ^) over time, as shown in the schematic diagram in Fig. S9. The competing adsorption of both dsDNA dyes was not observed, although PO-PRO-1 diffusion is assumed to be higher due to its smaller molecular size compared to PI^6^,^45^. PO-PRO-1 is not considered problematic for use in single-cell studies, as shown by Wlodkovic *et al*. or for use at higher concentrations with mammalian cells^20^ and has been tested in *B. subtilis* (Fig. S3).

The other non-invasive counterstain method presented here for single-cell-death studies employed non-toxic fluorogenic esterase substrates (Fig. S3). CgAM is taken up as an esterase substrate into the cytosol and secreted by active efflux pumps or sequestered in either vacuoles or in the cytosol if ATP is depleted^46^,^47^. Aged cells sequestering calcein green (CALg) in their vacuoles were assumed to have lost their V-ATPase activity prior to achieving a PI^ + ^state due to the loss of organelle function. As with PO-PRO-1, apoptotic-like phenotypes appeared as CALg^ + ^before presenting as CALg^ + ^/PI^ + ^(Fig. S8).

**Figure 5:**
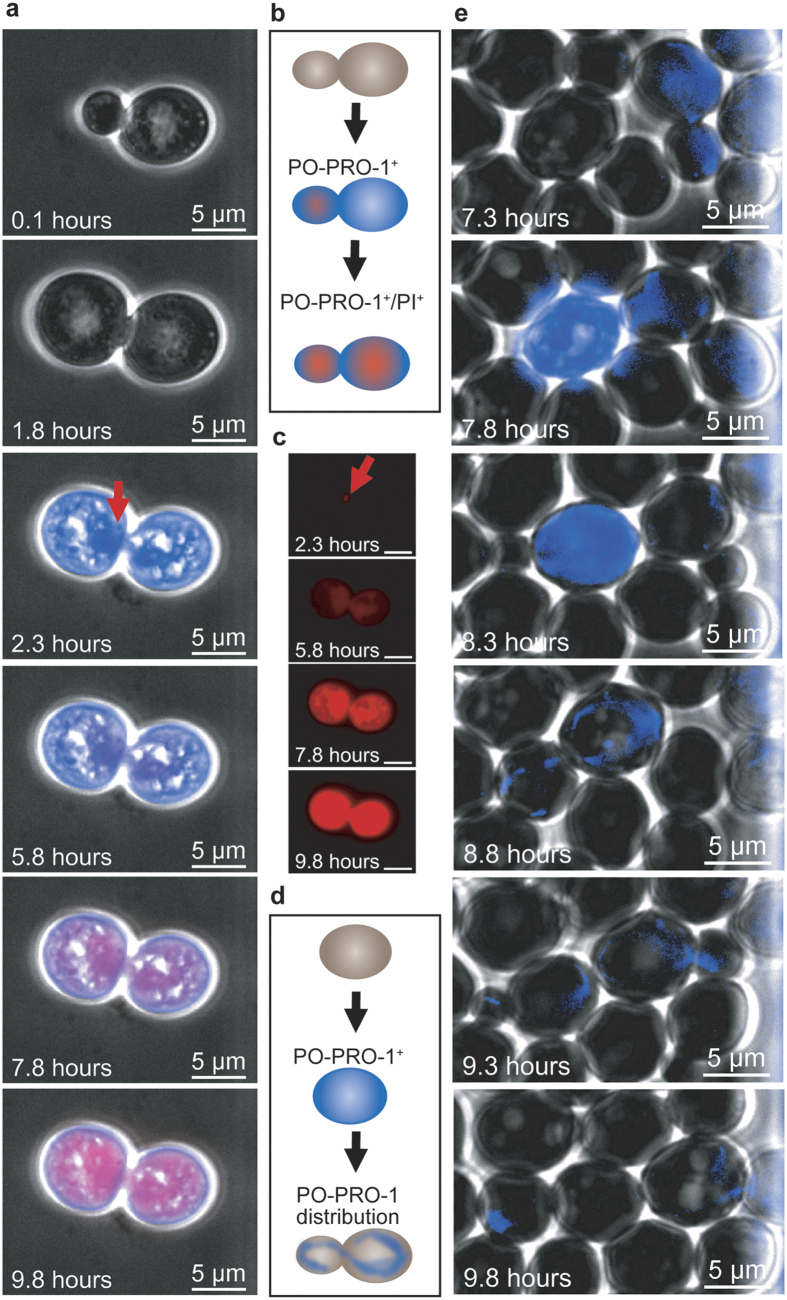
Budding and cell death. (**a**) A budding yeast cell was injured at the budding neck (marked with red arrow) and subsequently died. (**b**) Schematic drawing of an apoptotic mother cell that lost its membrane potential at the same time as its bud cell, which was injured near the budding neck. Both cells were PO-PRO-1^+^/PI^+^. (**c**) The pictures indicate that spatiotemporally resolved PI fluorescence diverges from the merged images in (**a**). **(d)** Schematic drawing of cell recovery due to cell budding. The cell became PO-PRO-1^+^ after membrane potential loss. The cell eventually initiated PCD while undergoing replication and proceeded with budding and actin-assisted DNA distribution (AT-rich regions appear fluorescent blue). **(e)** A cell exhibiting apoptotic-like behaviour followed by recovery due to budding is marked with white arrows. PO-PRO-1 fluorescence increased and was maintained for one hour before budding was initiated. DNA passage into the daughter cell was observable due to the presence of the DNA indicator PO-PRO-1. Mother and daughter yeast cells proceeded to budding followed by the dilution of fluorescence in the next filial generation.

We distinguished between necrotic-like phenotypes by employing non-toxic dual staining (Fig. S8 and S9) to observe cells exhibiting the hallmarks of ageing (Fig. S8 and S9) or undergoing lethal autophagy (Fig. S8 and S9), the apoptosis of budding mother or zygote cells (Fig. S9), and shmoo mating with aged cells (Fig. S9). PCD induction was primarily observed in the budding descendants of progenitor yeast cells derived from stationary, nutrient-deprived pre-cultures. However, dynamic live-cell analysis with PI and PO-PRO-1 permits hours of time-resolved monitoring of yeast fission and the subsequent loss of membrane potential indicated by PO-PRO-1 loading prior to PI uptake, as shown in Fig. 5a,b. The spatial resolution of PI fluorescence revealed injury close to the budding neck (Fig. 5a,c, marked with red arrows), where the replicated DNA from the mother is passed to the daughter cell. The mother and daughter cells were still connected and shared the same fate: the initiation of death.

In contrast, the absence of PI fluorescence in a PO-PRO-1^ + ^cell and the loss of PO-PRO-1 fluorescence are indicative of pre-apoptotic cells able to undergo growth recovery (Fig. 5d,e, Video S7) among rapidly growing cells. PO-PRO-1 is a very selective indicator of double-stranded DNA that is not sequestered in a manner consistent with other cell components^45^. PO-PRO-1 fluorescence decreased between two imaging time points, which cannot be explained by bleaching, and this property was passed on to emerging daughter cells during meiosis (Fig. 5e).

Cells seeded after famine conditioning in a pre-culture shaking flask were partly growth-inhibited but were not PI^ + ^. Based on PO-PRO-1 staining, these growth-inhibited yeast cells formed small circular blue fluorescent patches localized close to the cell membrane. Extrachromosomal ribosomal DNA circles (ERC) are indicative of the replicative ageing of yeast cells^48^. Interestingly, not all cells were stained, similar to early apoptotic cells undergoing DNA fragmentation.

**Figure 6:**
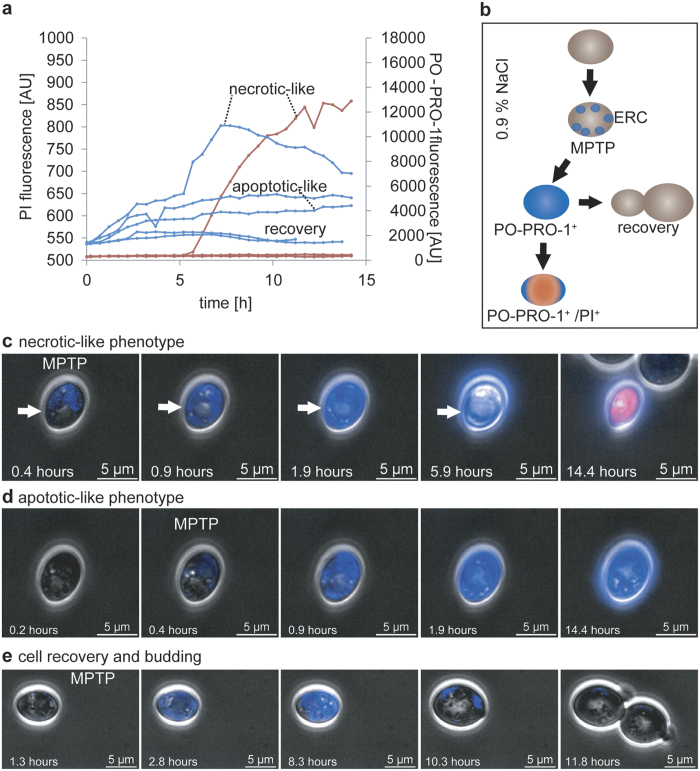
Stress-triggered cell rescue via membrane permeability transmission pore formation. (**a**) PI and PO-PRO-1 fluorescent traces in cells that initially demonstrated local PO-PRO-1 staining at the cell membrane due to mitochondrial permeability transition pore (MPTP) formation are shown. Spatiotemporal resolution of the intracellular fluorescence revealed a necrotic-like phenotype, an apoptotic-like phenotype, and cell resuscitation as explained in (**b**) and depicted in (**c–e**). (**b**) After exposure to nutrient starvation conditions, single cells were observed to be permeable, to a certain extent, to dye loading with PO-PRO-1 due to its small molecular size. Blue fluorescent patches near the cell membrane were assumed to indicate extrachromosomal rDNA circles (ERC), which are thought to influence the life span and chronological ageing of yeast. This leads to apoptosis (PI^−^/PO-PRO-1^+^) and necrotic-like apoptosis (PI^+^/PO-PRO-1^+^). Apoptotic-like cells were capable of recovery and division. **(c)** An aged cell with an enlarged vacuole (white arrow) exhibited initial partial membrane permeability to PO-PRO-1 due to MPTP formation. This was followed by loss of membrane potential. The necrotic-like phenotype was characterized by decreased size, PI^+^ staining, and the induction of death over several hours. Size reduction and a reduced death period are characteristic of necrotic-like apoptosis. **(d)** Cells that were partially permeable to PO-PRO-1 likely formed MPTPs and lost their membrane potential. The fluorescence exhibited by this apoptotic-like phenotype increased over time. **(e)** Cells that undergo MPTP formation are capable of recovery, as shown here. The cell internalized PO-PRO-1 and remained fluorescent for more than 8 h before the fluorescence disappeared from the majority of intracellular areas. This reduction in fluorescence was followed by budding and cell division.

PO-PRO-1 is a much smaller molecule than PI and enters the cells due to perturbations in mitochondrial permeability. Mitochondrial permeability transition plays a role in MPTP formation, leading to necrosis (Fig. 6c) or apoptosis (Fig. 6d)^49^. Mitochondrial permeability transition is the reversible differentiation of mitochondria resulting in increased permeability to solutes smaller than 1500 Da, depolarization, swelling, and ATP production^49^. Cells are able to recover via the microautophagy of dysfunctional mitochondria (Fig. 6e)^50^. We observed three phenotypes distinguishable based on their PO-PRO-1 fluorescent traces: a necrotic-like phenotype, an apoptotic-like phenotype, and a resuscitative phenotype (Fig. 6).

## Discussion

We demonstrated a one-step, non-invasive, dynamic PI staining method inside a microfluidic cultivation device that supported the real-time observation of cell death events among prokaryotic and eukaryotic cells. Non-toxic counterstains (CALv, CALg, and PO-PRO-1) were selected to facilitate real-time testing for survivor cells or to obtain additional information regarding cell status. The continuous supply of fluorochromes in media ensured optimal distribution in dense cell cultures over the experimental period. Thus, optimal fluorochrome concentrations could be realized in the μM or smaller range, avoiding non-specific cell staining that occurs due to fluorochrome uptake at high concentrations. Furthermore, no experimental disruptions due to sampling were necessary. Cellular-triggered differentiation and subsequent death were observed in real time for selected cells and colonies under specific cultivation conditions of interest. This permitted observation of the development of rapid cell death phenotypes (*e.g.*, lysis) or more complex PCD occurring over time under low ATP consumption conditions.

PI staining is among the most widely used methods to detect cellular death. However, this staining method is discussed very inconsistently in the literature^1^,^2^,^3^,^4^,^5^,^6^. Some researchers have described contradictory staining results, and the conventional staining protocol is prone to error during certain steps and using parameters, such as the dye incubation time, wash buffers, and dye concentration^3^. The often-mentioned occurrence of false PI^ + ^cells may be attributable to the use of dye concentrations that are too high. Although considered impermeable, viable cells may be stained by diffusion-driven uptake if the concentration gradient at the outer cell wall boundary is sufficiently large.

Although a longer staining duration increases PI^ + ^cell numbers^1^, low-level, optimized PI perfusion is non-toxic for use with microfluidic cultivation. The effects of longer staining duration may be explained by the on-going death of moribund cells during endpoint sample staining due to the bactericidal impacts of storage, nutrient deprivation, osmotic shock, counterstain toxicity, or high PI concentrations, given that the toxicity of assay conditions is generally not taken into account. However, viable cell numbers have also been overestimated due to the stronger binding of SYTO 9 to DNA binding sites in comparison to PI^2^,^6^.

We determined that a constant PI concentration of 0.1 μM was sufficient for bacteria and yeast. A concentration of 10 μM PI, which corresponds to 6.7 μg/mL, results in a concentration gradient between the inside and outside of viable Gram-positive cells that facilitates partial PI intrusion. However, yeast staining using 6 μg/mL PI have been reported to provide inconsistent staining results^1^. Previous studies have employed batch staining approaches with PI concentrations ranging from 4 μM to 500 μM for Gram-negative *E. coli*^11^,^23^, 3 μM for Gram-positive *Mycobacterium smegmatis*^17^, and 30 μM to 149.6 μM for *S. cerevisiae*^29^,^51^. Continuous PI contact with cancer cells has been achieved with concentrations ranging from 0.37 μM to 3 μM PI in microfluidic devices^20^,^30^.

Furthermore, we demonstrated the presence of segmented cells with a PI^ + ^dead cell pole and an antibiotic-tolerant, metabolically active cell pole attributable to the incomplete cell division of stressed cells caused by the addition of antibiotics. Extrinsic growth perturbation (as shown in this study for *C. glutamicum* utilizing bacteriostatic antibiotics) as well as intrinsic stress^52^ lead to growth arrest and the inhibition of cell division. In contrast to microscope-based analytical systems, FACS analysis possesses the disadvantage that aggregated PI^−^ and PI^ + ^cells are considered PI-stained cells^1^. Thus, if a surviving PI^−^ cell is attached to a PI^ + ^cell or the cell pole is recovered, misinterpretations are possible.

The continuous addition of counterstains together with PI is challenging if toxicity and growth impairment must be avoided. We found CAM derivatives and the membrane potential-indicating stain PO-PRO-1 to be suitable for non-toxic counterstaining in combination with PI. These stains are appropriate for live-cell imaging applications^32^,^45^,^53^ and permit the indication of residual cell functionality in survivor cells or cells in the early stages of PCD. In particular, the temporal resolution of single-cell dye uptake is crucial for cell death analyses.

The use of a microfluidic device allows for an integrated approach involving non-toxic dynamic PI staining during cell cultivation. Detailed examination of cellular PI uptake as well as time-resolved observation of phenotypic differentiation in dying single cells were possible in treated cells, in contrast to the reference cells that proceeded growth. Here, we present the novel use of a microfluidic cultivation device for the time-resolved analysis of cellular survival and studies of PCD employing fluorescent dyes to detect intracellular changes.

The dynamic analysis of single-cell viability comprises more than the differentiation of dead and alive cells. Temporal differentiation permitted intermediate states and intermediate changes in cell status to be distinguished (alive to dead or lysed, moribund to resuscitated, alive to autolysed, dead to lytic decay). Our single-cell fluorescence analysis is particularly relevant for studies examining phenotypically heterogeneous and spontaneously occurring cell survival or lethal cell differentiation. Thus, our method has the potential to contribute to studies of autolysis, autophagy, antibiotic tolerance, spontaneous resistance, epigenetic triggered cell differentiation, membrane integrity, drug testing, medical care and many other areas of research. Its broad applicability is not only limited to typal specifications and may also inspire applications involving biofilms, mammalian cells or thin tissue layers. Our dynamic live/dead staining method can be adapted to other existing media-perfused microfluidic cultivation devices for single-cell fluorescence imaging if the duration of cellular death or survival is of interest. Such analyses will allow lingering questions to be answered during molecular biological studies. Additionally, our approach can be integrated into fluorophore expression studies through the use of multiplexed imaging.

## Materials and Methods

### Bacterial cultivation and media

The materials for media preparation were provided by Carl Roth, Karlsruhe, Germany, unless otherwise stated. *C. glutamicum* ATCC 13032 wild-type cultures were pre-cultured with brain heart infusion medium (BHI, BD, Heidelberg, Germany) at 30 °C overnight. Then, the primary shaking culture was inoculated in BHI medium, or additional pre-culture was performed using minimal medium (CGXII containing 4% glucose (w/v), described by Keilhauer *et al*.) to inoculate the primary shaking culture with the same minimal medium^54^. Cells from the primary culture were injected into the microfluidic device for single-cell cultivation and perfused with medium as indicated. *B. subtilis* 168 was cultivated in BHI at 37 °C in shaking flasks and in the microfluidic device. *V. harveyi* DSMZ 6904 was grown in marine broth 2216 (BD, Heidelberg, Germany) at 30 °C (shaking cultures) and 28 °C (microfluidic cultivation). *M. luteus* DSMZ 14234 shaking cultures were grown in a nutrient solution consisting of 5 g/L peptone and 3 g/L meat extract, adjusted to pH 7, and incubated at 30 °C before cultivation at 28 °C in the microfluidic device. *E. coli* MG1655 was cultivated in lysogeny broth (LB) containing 5 g/L yeast extract, 10 g/L peptone, and 10 g/L NaCl at 37 °C in shaking cultures and during microfluidic cultivation. Chemically competent *E. coli* BL21CodonPlus(DE3)-RIL cells were transformed with pET28b(*pezTΔ*C242), pET28b(*pezTΔ*C242(*D66T*)), or pET28b(*pezA/pezT*) (kindly provided by A. Rocker and A. Meinhart, MPI Heidelberg, Germany). *E. coli* BL21CodonPlus(DE3)-RIL shares high genomic similarity with *E. coli* MG1655 and was used in lieu of MG1655 due to its improved codon usage and induction properties for heterologous protein expression^55^. After transformation, mutants were cultivated at 37 °C overnight on LB media agar plates with 50 μg/mL kanamycin and 34 μg/mL chloramphenicol. The next day, single colonies were selected and transferred to LB medium containing 50 μg/mL kanamycin and 34 μg/mL chloramphenicol. Before cell seeding in the microfluidic device, shaking cultures were incubated at 37 °C until an optical density higher than 0.1 was reached. Protein expression during microfluidic cultivation was induced by changing the LB perfusion medium containing 50 μg/mL kanamycin and 34 μg/mL chloramphenicol to an LB medium containing 100 μM IPTG in addition to both antibiotics^36^. Commercially available compressed baker’s yeast (*S. cerevisiae*) (UNIFERM GmbH & Co.KG, Werne, Germany) from the supermarket was dissolved in YPD medium (20 g/L peptone, 10 g/L yeast extract, and 20 g/L glucose) and pre-cultivated 48 h prior to the primary cultivation for microfluidic device inoculation. Starved cells were cultivated prior to microfluidic cultivation with fresh YPD medium for 48 h in YPD medium or 0.9% NaCl solution (w/v).

### Dynamic staining

PI (Carl Roth, Karlsruhe, Germany) stock solution (20 mM) was prepared and dissolved in sterile water. PI was added to the media perfused through the microfluidic device. An end concentration of 0.1 μM was used for dynamic viability staining in the microfluidic device unless otherwise stated. Counterstaining with CvAM (Life Technologies GmbH, Darmstadt, Germany) was performed for *C. glutamicum* as described in detail elsewhere^32^. CgAM (Life Technologies GmbH, Darmstadt, Germany) and calcein blue acetoxymethyl ester (CbAM, Life Technologies GmbH, Darmstadt, Germany) were used as sequestering agents for the dynamic staining of *S. cerevisiae* cells. All calcein acetoxymethyl esters (CAMs) were prepared as fresh 2.5 mg/mL stock solutions in water-free DMSO (Carl Roth, Karlsruhe, Germany) directly before use. In addition to 0.1 μM PI, 46 μM CvAM, 60 μM CbAM, or 28 μM CgAM was dissolved in the indicated perfusion media. Alternatively, 2 μM PO-PRO1 (Life Technologies GmbH, Darmstadt, Germany) was added to the PI-containing perfusion medium to indicate cells without membrane potential. Radical oxygen species (ROS) formation is an indicator of phototoxicity^56^. Reduced dihydroxycalcein-acetoxymethyl ester (DHCAM, Life Technologies GmbH, Darmstadt, Germany) was used to detect ROS. Dye adsorption or absorption by PDMS was not observed as determined by LogP values and molecular size.

### Microfluidic device and time-lapse imaging

A microfluidic device platform was used for microbial cultivation and single-cell analysis. This device is described in detail by Kohlheyer and co-workers^31^. The microfluidic device harbours microstructures with dimensions to ensure the growth of several hundred cells in a monolayer in the perfusion culture. Different media used were infused with a high-precision syringe pump (neMESYS, Cetoni GmbH, Korbussen, Germany) at a rate of 300 nL/min. A constant cultivation temperature was ensured by an incubation chamber (PeCon GmbH, Erbach, Germany).

The microfluidic device was installed on an inverted epifluorescence microscope (TI-Eclipse, Nikon GmbH, Düsseldorf, Germany), which was equipped with a motorized stage (Nikon GmbH, Düsseldorf, Germany), high-speed charge-coupled device (CCD) cameras (Clara DR-3041 and Neo sCMOS, Andor Technology Plc., Belfast, United Kingdom), the Nikon Perfect Focus System (PFS, Nikon GmbH, Düsseldorf, Germany) for thermal drift compensation, and a Plan Apo 100 Oil Ph3 DM objective (Nikon GmbH, Düsseldorf, Germany). For phase contrast images, a cooled LED light source (at 3–8% of total intensity) was used. Epifluorescence illumination was performed with a mercury light source (Intensilight, Nikon GmbH, Düsseldorf, Germany, set to 1/32 of total intensity and additionally reduced 1/8 by filter settings). Fluorescent time-lapse imaging was carried out based on the growth rate and photosensitivity of the species, described as follows: every 8 min for *C. glutamicum*, every 5 min for *E. coli*, *B. subtilis*, and *V. harveyi*, every 30 min for *S. cerevisiae*, and every 60 min for *M. luteus*. A TRITC filter (EX 540/25 nm, DM 565 nm, BA 605/55 nm, Nikon GmbH, Düsseldorf, Germany) and a Texas Red filter (EX 540–580 nm, DM 595 nm, BA 600–660 nm, AHF Analysentechnik AG, Tübingen, Germany) were employed for PI signal imaging. CvAM and CbAM were analysed with a DAPI filter (EX 540–380 nm, DM 400 nm, BA 435–485 nm, Nikon GmbH, Düsseldorf, Germany). CgAM was excited with a FITC filter (EX 465–495 nm, DM 505 nm, BA 515–555 nm, Nikon GmbH, Düsseldorf, Germany). A CFP HC filter set (EX 438/24 nm, DM 458 nm, BA 483/32 nm, Nikon GmbH, Düsseldorf, Germany) was used for the PO-PRO1 dye.

### Data analysis

Time-lapse imaging data from antimicrobial-treated *C. glutamicum* cells (Fig. 2 and Fig. S2), yeast cells (Fig. 1), and *E. coli* BL21(DE3) CodonPlus(DE3)-RIL cells transformed with pET28b(*pezTΔ*C242), pET28b(*pezTΔ*C242(*D66T*), or pET28b(*pezA/pezT*) (Fig. 4) were analysed using the counting option of the NIS-Elements software program to enumerate total cell numbers and PI^+^ cells with identical LUT settings. The analysis of prokaryotic imaging data, shown in Figs 1 and 3, and Fig. S3–S7, was facilitated by a user-specialized workflow run constructed as an ImageJ/Fiji plugin^57^. Cell identification was performed using a segmentation procedure tailored to detect individual bacterial cells in crowded populations. All frames were checked manually to remove artefacts as well as to identify segmentation and cell identification failures. Yeast cells were segmented manually using ImageJ. Identified cells were subsequently linked throughout image sequences by implementing an adapted single-particle tracking approach with TrackMate^58^. Image analysis permitted the extraction of measurable quantities of individual cells, (*i.e.*, mean fluorescence) as shown in Figs 3 and 6, and Fig. S4. Finally, data sets derived from *C. glutamicum* treated with antibiotics were processed to generate individual single-cell traces over time using the analysis and visualization software Vizardous, as recently described in detail^59^.

### Cell classification criteria

PI intrusion of cell wall-compromised cells was indicated by red fluorescence. PI^+^ cells were considered dead. Cells were defined as PI^+^ if the PI fluorescence increased drastically between two frames or if the initial basal fluorescence signal was increased by more than 5% and demonstrated an increasing trend in subsequent time-lapse images until equilibrium was achieved. CAM conversion to fluorescent CAL indicated enzymatic activity in cells or cell organelles (yeast). Uninhibited growing *C. glutamicum* cells exhibited moderate fluorescence (CAL^+^) due to the partial efflux of CAL, whereas cells with reduced metabolic activity exhibited increased CAL fluorescence (CAL^++^) in comparison to CAL^+^ cells^32^. Yeast cells with reduced vATPase activity retained CAL in their vacuoles. A reduction in cellular enzymatic activity was a criterion for reduced cell activity and cell survival. Cells that lost their membrane potential or had cell membrane injuries underwent PO-PRO-1 intrusion, indicated by blue fluorescence. PO-PRO-1^+^ cells were considered dying or dead, but recovery was observed for yeast cells. Cells were defined as PO-PRO-1^+^ if the PO-PRO-1 fluorescence increased drastically between two frames. Lysed cells were non-fluorescent due to the loss of molecule retention, observed as pale cells in phase contrast images, or cell debris disintegration.

## Additional Information

**How to cite this article**: Krämer, C. E. M. *et al*. Time-resolved, single-cell analysis of induced and programmed cell death via non-invasive propidium iodide and counterstain perfusion. *Sci. Rep.* **6**, 32104; doi: 10.1038/srep32104 (2016).

## Supplementary Material

Supplementary Information

Supplementary Video S1

Supplementary Video S2

Supplementary Video S3

Supplementary Video S4

Supplementary Video S5

Supplementary Video S6

Supplementary Video S7
