# Supplementary material for: Time-resolved, single-cell analysis of induced and programmed cell death via non-invasive propidium iodide and counterstain perfusion
Source: Sci Rep. 2016 Sep 1;6:32104. doi: 10.1038/srep32104 (PMC5007472; doi:10.1038/srep32104)
Supplement: Supplementary Information [file srep32104-s1.pdf]

Supplementary Information

Time-resolved, single-cell analysis of induced and programmed cell death via non-invasive propidium iodide and counterstain perfusion

Christina Krämer[1], Wolfgang Wiechert[1], Dietrich Kohlheyer[1,*]

[1]IBG-1: Biotechnology, Forschungszentrum Jülich GmbH, Jülich, Germany

*Correspondence:
Jun. Prof. Dr. Dietrich Kohlheyer
IBG-1:Biotechnology
Forschungszentrum Jülich
52425 Jülich
Germany
E.-mail: d.kohlheyer@fz-juelich.de
Phone: +49 2461 612875

Supplementary Abstract

The dynamic staining of living microorganisms under different stress situations was analysed in a microfluidic perfusion device. This microfluidics approach advanced conventional staining through the use of a reduced propidium iodide concentration that did not affect single-cell growth. Simultaneous validation utilizing non-toxic counterstaining and spatiotemporal resolution permitted us to analyse the emergence of lethal cell wall damage, cell lysis with DNA discharge, or the bipolarity of cell-division–inhibited cells. Data analysis has not yet been fully automatized.

Supplementary Information

Microfluidic environment

The microfluidic device employed for our dynamic staining studies permits control of the bacterial microenvironment as demonstrated experimentally and through CFD simulations in previous studies [1–4]. The cultivation chamber arrays lack multiple channel inlets for sink and supply settings as well as a gradient mixer in the upstream region, in contrast to previous designs [5,6].

Continuous medium supply and nutrient renewal are present in the described microstructures. The microstructures and the environmental control of the microfluidic perfusion system used in this work were described in detail previously [1–3]. A continuous nutrient supply and waste-product removal were implemented in studies examining the roles of glucose, a major carbon source, as well as a minor carbon source (an iron chelator) during *C. glutamicum* cultivation [3,4].

For our dynamic staining studies, chemicals and dyes were selected based on their hydrophilic characteristics, water solubility, and LogP values (LogP < 2). The cultivation chambers were randomly chosen and analysed. Clustering of fluorescence data was not observed in the transverse direction of the microchamber arrays for multiple parallel channels, in the flow

direction of the microchambers, or over time for any experiment. Final single-cell fluorescence values of antibiotic-treated *C. glutamicum* cells are shown in a directional distribution for the microchamber array (Fig. S1).

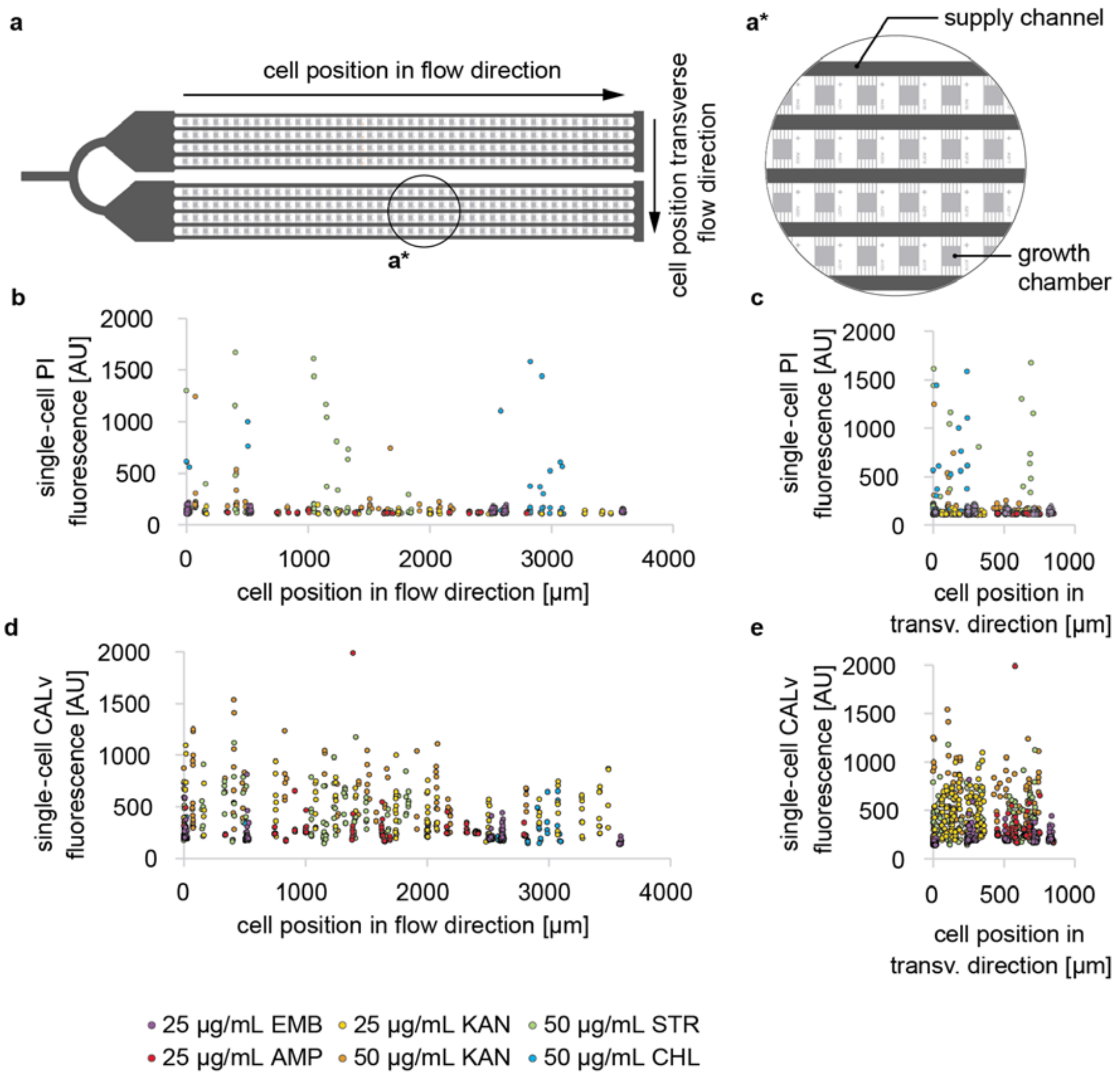

Figure S1 Single-cell distribution and fluorescence of PI and CALv in antibiotic-treated *C. glutamicum* ATCC 13032 cells in the microfluidic chamber array. (a) Microfluidic channel network and growth chamber array. **(a*)** Enlarged view of the growth chamber array. Final distributions of single-cell fluorescence in the flow direction for **(b)** PI and **(d)** CALv and in the transverse direction for **(c)** PI and **(e)** CALv.

Supplementary Results

Dynamic PI staining validation

Dynamic PI staining was validated by adding a disinfectant to the perfusion medium. Conventionally, PI viability analyses are validated with cell suspensions treated with isopropanol or ethanol [7,8]. Both isopropanol and ethanol are able to penetrate the device material PDMS and diffuse from channels containing high organic solvent concentrations to neighbouring solvent-free channels through the wall of the microfluidic device [9].

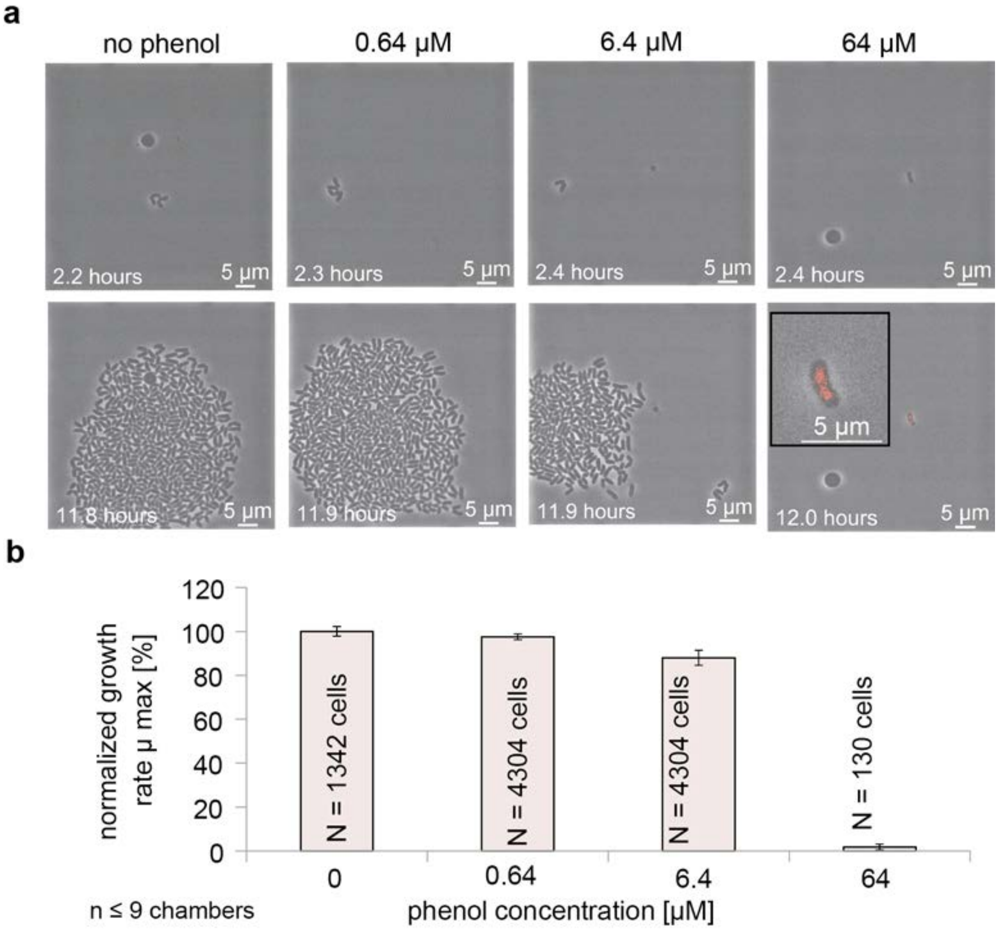

Figure S2 Toxicity test with *C. glutamicum* ATCC 13032 and phenol. Three phenol concentrations (0.64 µM, 6.4 µM, and 64.0 µM) were continuously perfused along with the medium (CGXII + 4 % GLC). **(a)** Images of the reference and all tested phenol concentrations are shown 2.2-2.4 h after the start of the experiment and after 11.8-12.0 h for comparison. **(b)** The growth rate, normalized to the untreated reference, was substantially reduced with 6.4 µM phenol, but 64.0 µM phenol almost completely halted cell growth and resulted in PI^+ cells after more than 8 h. Final total cell numbers are given by N. Standard deviation is indicated by error bars.

Therefore, we used phenol at concentrations ranging from 0.64 µM to 64 µM in CGXII + 4 % GLC, rather than ethanol or isopropanol, to avoid material interactions. A phenol concentration of 6.4 µM or less was sub-lethal, whereas 64 µM was lethal for all imaged cells (N = 130 cells) after 10 h. Only two cells exhibited marginal cell growth before cell division stopped. After 10 h of treatment with 64 µM phenol, all *C. glutamicum* cells exhibited red PI fluorescence (exemplary cell shown in Fig. S2).

A "gold standard" for the non-disintegrative cell death of a broad variety of microorganism is difficult to define. For aerobic and facultative anaerobic microorganisms, we tested the addition of a lethal sodium cyanide concentration (50 mg/mL) to inhibit oxidative metabolic reactions in combination with the membrane potential loss indicator PO-PRO-1 in the cultivation media for *C. glutamicum*, *M. luteus*, *B. subtilis*, *E. coli*, *V. harveyi*, and *S. cerevisiae*. After membrane potential loss was indicated for a substantial number of cells, dynamic PI staining was initiated. Gram-positive bacteria had a signal-to-background ratio (S/B) between 1.78 and 1.84. Gram-negative bacteria demonstrated an S/B ranging from 2.36 to 2.62, and the analysed yeast strain had an S/B of 4.73 (Fig. S3 a).

The signal-to-noise ratio (S/N) varied by typal characteristics (cell size, cell wall thickness, DNA content), ranging from 37.4-fold (*E. coli*) up to 901.5-fold (*S. cerevisiae*) (Fig. S3 a). The cyanide incubation time reflected the cyanide resistance of individual cells (Fig. S3 b). For the tested Gram-positive bacteria and the yeast strain, the dead-cell ratios were identical. *E. coli* and *V. harveyi* achieved 84.0 % and 110.7 % dead cells (PI^+), and some cells lost their membrane potential due to substantial cell lysis accompanied by DNA loss (*E. coli*), cell disintegration (cell loss) (*V. harveyi*) and ongoing cell death (*V. harveyi*) (Fig. S3 b).

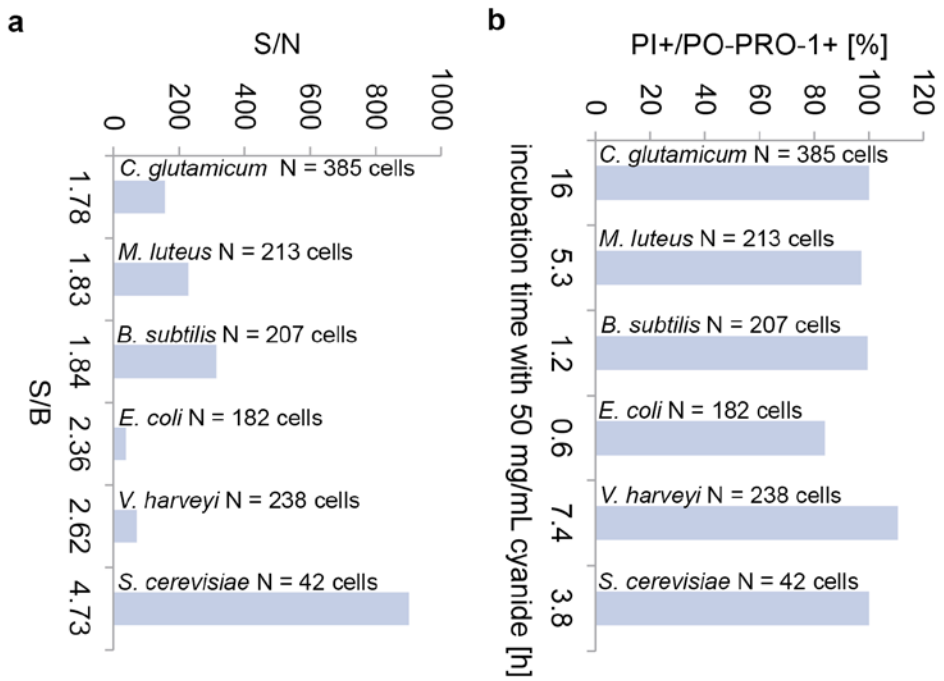

Figure S3 Validation of dynamic PI staining. Final total cell numbers are given by N. **(a)** Signal-to-noise ratio over signal-to-background ratio. **(b)** Ratio of PI^+ cells to PO-PRO-1^+ cells during incubation with a lethal cyanide concentration required for substantial cell death.

Instantaneous cell death monitoring and non-toxic counterstaining

A counterstain was employed to ensure that nongrowing, PI-negative (PI^-) cells were intact and viable cells exhibiting growth inhibition. A noninvasive counterstain for microfluidic cultivation must meet several criteria, such as a sufficient signal-to-noise ratio, no inhibition of growth and division (toxicity), cellular uptake and fluorochrome containment, no adhesion or penetration of the channel wall material, stability during experiments, and no overlap of excitation or emission spectra with PI.

SYTO 9, which is used in the BacLight™ assay, stains the DNA of all cells. Given that SYTO 9 impairs cell growth during microfluidic cultivation and has been reported to be somewhat toxic [10], it was not used. Acridine orange is considered harmful because it induces a 40 % growth rate reduction in *C. glutamicum* due to DNA-dye interactions. Furthermore, CTC was tested with *E. coli* MG1655 and found to inhibit cell growth (data not shown), which is consistent with findings reported in the literature [11].

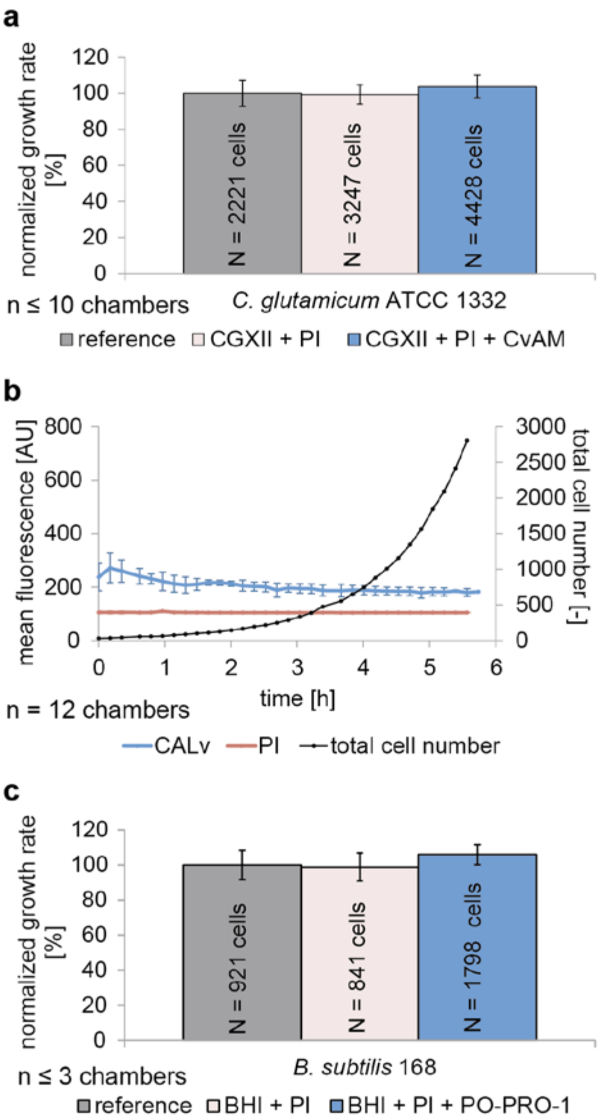

Figure S4 Comparison of bacterial growth under reference conditions in growth medium, growth medium with PI, and growth medium with PI and counterstain. (a) The mean growth rates of at least 10 colonies cultivated with minimal medium (CGXII + 4 % glucose (w/v)) (reference), minimal medium with 0.1 µM PI, and minimal medium with 0.1 µM PI and 46 µM CAM demonstrate that the addition of PI and CAM is not detrimental to *C. glutamicum* cell growth. Final total cell numbers are given by N. Standard deviation is indicated by error bars. **(b)** The mean fluorescence values of PI (red) and the fluorogenic substrate CvAM, used as a counterstain (blue), are shown along with the total cell numbers of all analysed cultivation chambers over time. The PI fluorescence did not change considerably over time because PI^+ cells only appear rarely under reference conditions. CvAM was taken up by all viable cells and converted to fluorescent CALv. Standard deviation is indicated by error bars. **(c)** The mean growth rates of at least 3 colonies cultivated with the complex medium BHI (reference), BHI with 0.1 µM PI, and BHI with 0.1 µM PI and PO-PRO-1 demonstrate that the addition of PI and CvAM is not detrimental to *B. subtilis* cell growth. Final total cell numbers are given by N. Standard deviation is indicated by error bars.

The fluorogenic group of esterase substrates of calcein-acetoxymethyl esters (CAM) met all criteria for *C. glutamicum*, as shown recently by Krämer et al. (2015) [12], as well as for

eukaryotic cells[13]. Violet fluorogenic CAM (CvAM) was taken up by *C. glutamicum* cells and converted by intracellular esterases to violet fluorescent calcein, which was secreted in an energy-dependent manner. Energy limitation is assumed to cause an increase in CvAM fluorescence due to intracellular accumulation[12].

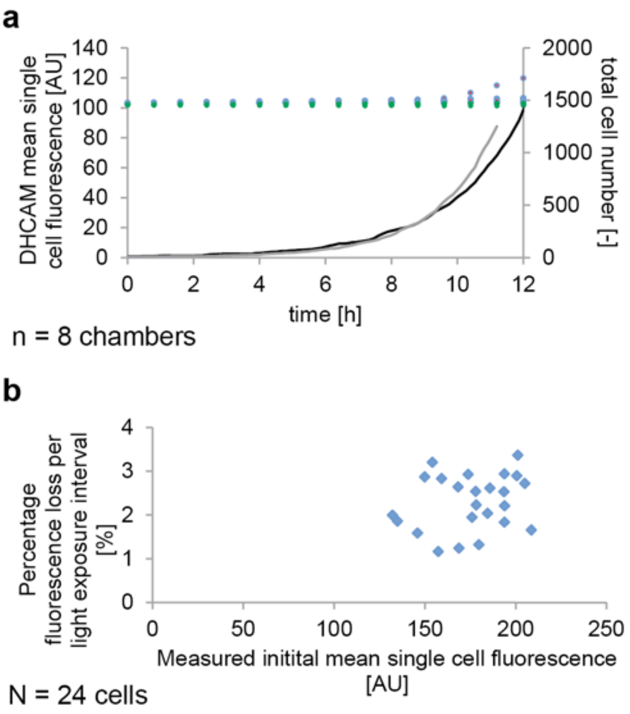

Figure S5 Phototoxicity and photobleaching analysis. (a) The phototoxicity of time-lapse imaging was tested with *C. glutamicum* and dihydrocalcein AM (DHCAM). DHCAM is taken up by *C. glutamicum* cells as CAM and converted intracellularly to DHCAM. DHCAM is oxidized to fluorescent calcein green if light-induced oxygen radicals are present in bacterial cells. The phototoxicities of phase-contrast time-lapse imaging and fluorescent time-lapse imaging (phase-contrast imaging and excitation of PI and CAM) were analysed. The total cell number is indicated by the grey (phase-contrast imaging) and black lines (phase-contrast and fluorescence imaging). The calcein green fluorescence of every measured cell is indicated by green (phase-contrast imaging) and red-blue dots (phase-contrast and fluorescence imaging). After 10 h of fluorescent time-lapse imaging, one cell out of more than 500 exhibited an increase in mean single-cell calcein green fluorescence. **(b)** The photobleaching ability of PI was evaluated using *C. glutamicum* cells pre-treated with ampicillin. The mean loss of single-cell PI fluorescence was found to be 2.3 ± 0.6 % per time-lapse imaging time point.

C. glutamicum growth was not impaired by the addition of PI or the simultaneous addition of PI and CvAM (Fig. S4 a). The mean CvAM and PI fluorescence profiles of *C. glutamicum* colonies are shown in Fig. S3 B. The formation of intracellular oxygen radical species due to phototoxicity during multiplexed fluorescent time-lapse imaging was tested with

dihydroxycalcein acetoxymethyl ester (DHCAM) as described previously [12]. DHCAM did not result in phototoxicity (Fig. S5 a). The loss of fluorescence upon repeated light exposure was found to be marginal for PI (Fig. S5 b), which was shown previously for CALv [12].

No intracellular calcein fluorescence of *E. coli* MG1655 or *B. subtilis* 168 was observable with continuous CvAM or green fluorogenic CgAM perfusion during microfluidic cultivation (data not shown). Therefore, PO-PRO-1 was tested with *B. subtilis* 168 and found to be a non-invasive counterstain for PI validation (Fig. S4 c). For *S. cerevisiae*, multiplexed fluorescent time-lapse imaging with PI, PO-PRO-1 and calcein derivatives was satisfactory for growth (Fig. S6).

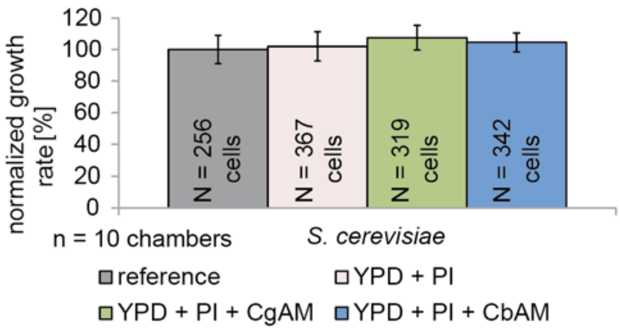

Figure S6 Comparison of yeast growth under reference conditions with growth medium, growth medium with PI, and growth medium with PI and counterstain. Green fluorescent calcein and blue fluorescent calcein were used as counterstains. The mean growth rates of at least 10 colonies were analysed for each condition. Final total cell numbers are given by N. Standard deviation is indicated by error bars.

Antibiotic treatment of *C. glutamicum* colonies

The mean PI fluorescence was found to be lower following the addition of cell wall synthesis inhibitors and 25 µg/mL KAN than with the addition of antibiotics that impair protein biosynthesis at a concentration at 50 µg/mL (Fig. S7). The mean calcein fluorescence was increased by the presence of an antibiotic (Fig. S7) relative to untreated colonies (Fig. S4 b). The striking variation in the standard deviation is primarily attributable to the microbial phenotypic heterogeneity of bacteria that die, are lysed, or temporally tolerate the presence of potentially fatal antibiotic concentrations. Furthermore, variation in the mean single-cell

fluorescence is likely attributable to differences in fluorochrome containment in antibiotic-affected cells.

The cells were enlarged by cell wall synthesis inhibitors, whereas EMB resulted in the asymmetric division of bulky cells with sharp poles, and AMP led to elongated cells with apical enlargement (Fig. 4 a-b). KAN and STR induced cell rounding and accounted for a fraction of the lysed cells (Fig. 3 c-e). KAN also initiated total cell disintegration. CHL halted cell division, resulting in segmented cells independently exhibiting metabolic activity or viability (Fig. 3 f).

The bactericidal antibiotics EMB and AMP inhibit cell wall synthesis and its repair mechanisms. Both antibiotics exerted a remarkable impact on *C. glutamicum* cells (Fig. 2 a, Fig. 3 a-b, and Fig. S7 a-b). Furthermore, both antibiotics resulted in reduced intracellular fluorescence compared to the reference (Fig. S4 b) and to other fluorescent cells treated with KAN, STR, or CHL (Fig. 3 c-f) due to the compromised intracellular retention of molecules by leaky cell walls.

The bacteriostatic antibiotics KAN, STR, and CHL inhibit bacterial protein biosynthesis. In addition to cell death and cell lysis (Fig. 2 b-c), these bacteriostatic antibiotics resulted in substantial increases in CALv fluorescence over time (Fig. S7 c-f) compared to non-treated reference cells (Fig. S4 b). Fractions of the total numbers of cells treated with KAN, STR, or CHL switched to an antibiotic-tolerant state accompanied by residual enzymatic activity (conversion of CvAM to CALv) and increased CALv retention (Fig. 2 and 3).

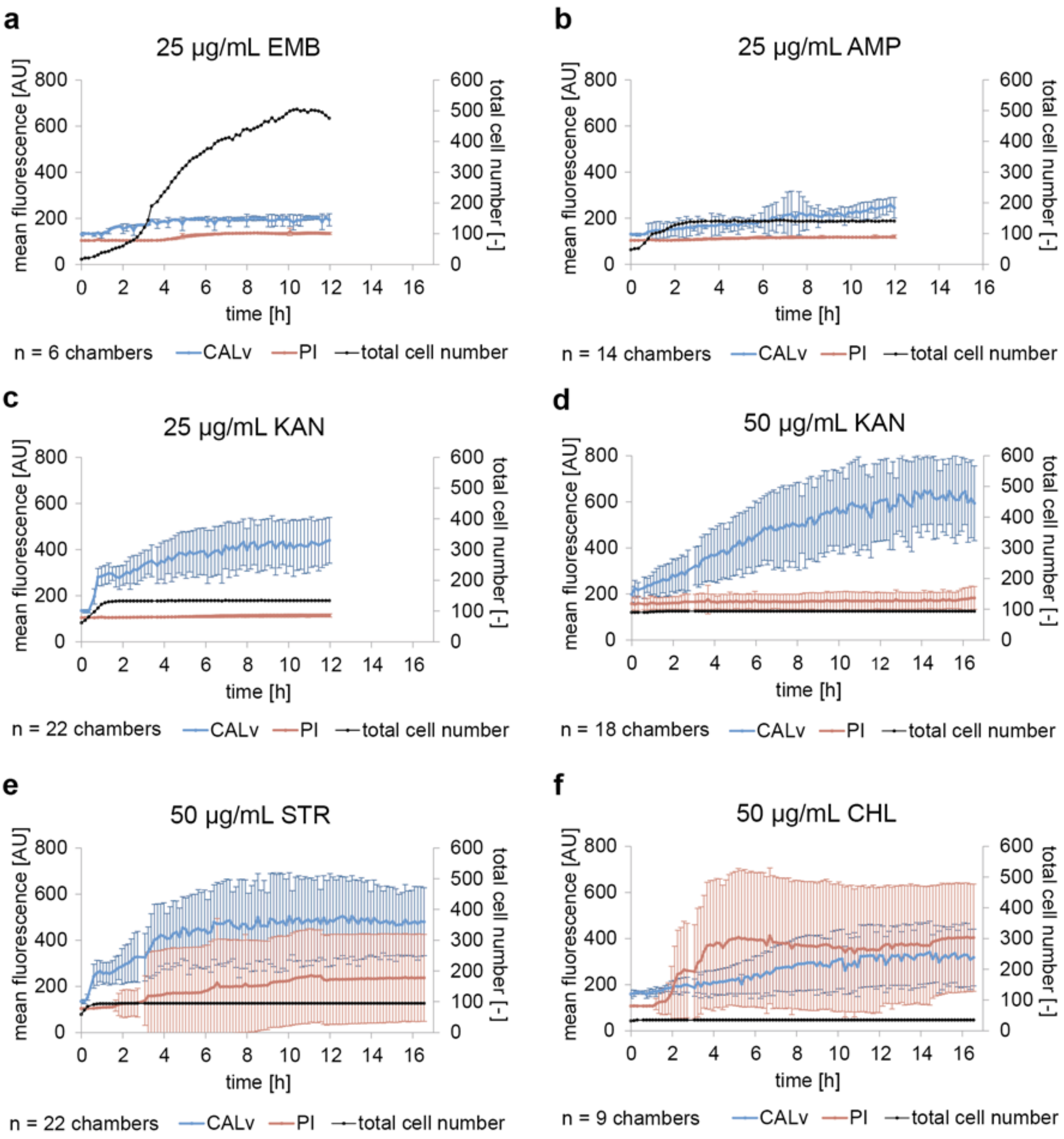

Figure S5 Continuous monitoring of cell wall integrity and metabolic activity of wild-type *C. glutamicum* ATCC 13032 colonies treated with different antibiotics using PI (red) and CvAM (blue). The total cell numbers of at least 6 colonies (black solid line) and the mean PI and CALv fluorescence values are indicated along with the standard deviation of all single cells over time. **(a)** Continuous treatment with the cell wall synthesis inhibitor EMB (25 µg/mL). **(b)** Continuous treatment with the cell wall synthesis inhibitor AMP (25 µg/mL). **(c)** Continuous treatment with 25 µg/mL KAN, which triggers mRNA misreading. **(d)** Continuous treatment with 50 µg/mL KAN, which triggers mRNA misreading. **(e)** Continuous treatment with 50 µg/mL STR, which triggers mRNA misreading. **(f)** Continuous treatment with 50 µg/mL CHL, which inhibits protein synthesis.

Differentiation of lethal phenotypes in yeast

Necrotic-like phenotypes immediately exhibited dual staining ($CALg^+/PI^+$ and $PO\text{-}PRO\text{-}1^+/PI^+$) (Fig. S9 a and Fig. S10 a). Leakage of green fluorescence was an indicator of

cell membrane rupture, as shown in Fig. S9 a, after 5.2 h of cultivation. CALg fluorescence increased for one hour prior to latent cell swelling and radical calcein release into the surrounding environment immediately before the cell became PI^+.

A fraction of cells exhibited remarkable vacuole enlargement, which has been shown to appear in aged yeast cells [14], followed by PI staining during the late phase of cultivation. Aged cells accumulated CALg in their vacuoles, which remained intact (Fig. S9 b and Video S4). Aged cells that produced enlarged vacuoles (Fig. S10 B, white arrow) were partially PO-PRO-1^+. During the late cultivation phase, aged cells became PI^+/PO-PRO-1^+ (Fig. S10 b).

In terms of autophagy in yeast, there are several cell recovery mechanisms, each with a known potential for failure [15]. Macroautophagy was rarely observed to end in fatality (Fig. S9 c, Fig. S10 c, and Video S3). Cells that immediately became PI^+ formed two separate vesicles (Fig. S9 c, white arrow) that were distinguishable based on interior CALg fluorescence and slight PI fluorescence surrounding the vesicle exterior. Both vesicles remained stable for more than 3 hours. After 4 hours, the vesicles disappeared following contact with the bud of a growing sibling.

Furthermore, shmoo-mediated mating between aged cells with enlarged vacuoles was observed. Shmooing and mating were not imaged during microfluidic cultivation. However, cells underwent shmooing or mating during pre-cultivation and were stochastically seeded in the microcultivation chamber for observation. Although zygote budding prior to cell death was observed (Fig. S9d and Video S5), shmoos lost their membrane potential after mating with cells that had previously lost their membrane potential. The cells formed a condensed, small vesicle (Fig. S10 d, white arrows, Video S6) inside the enlarged vacuole concurrent with slight PI fluorescence surrounding the vacuole. The vacuole was likely under pressure because bursting was observed. PI fluorescence did not appear inside the vacuoles, resulting in the heterogeneous distribution of fluorescence inside the shmoo-cell aggregate.

Additionally, the autophagy of a budding mother cell was observed (Fig. S10 C, white arrow). The mother and daughter cells both lost their membrane potential simultaneously. The cells did not divide terminally because first the daughter and then the mother cell became PI^+. The lysosome exhibited dual fluorescent signals (PO-PRO-1^+/PI^+), indicating the presence of DNA content. The vesicle remained stable for more than ten hours with minimal fading of fluorescence.

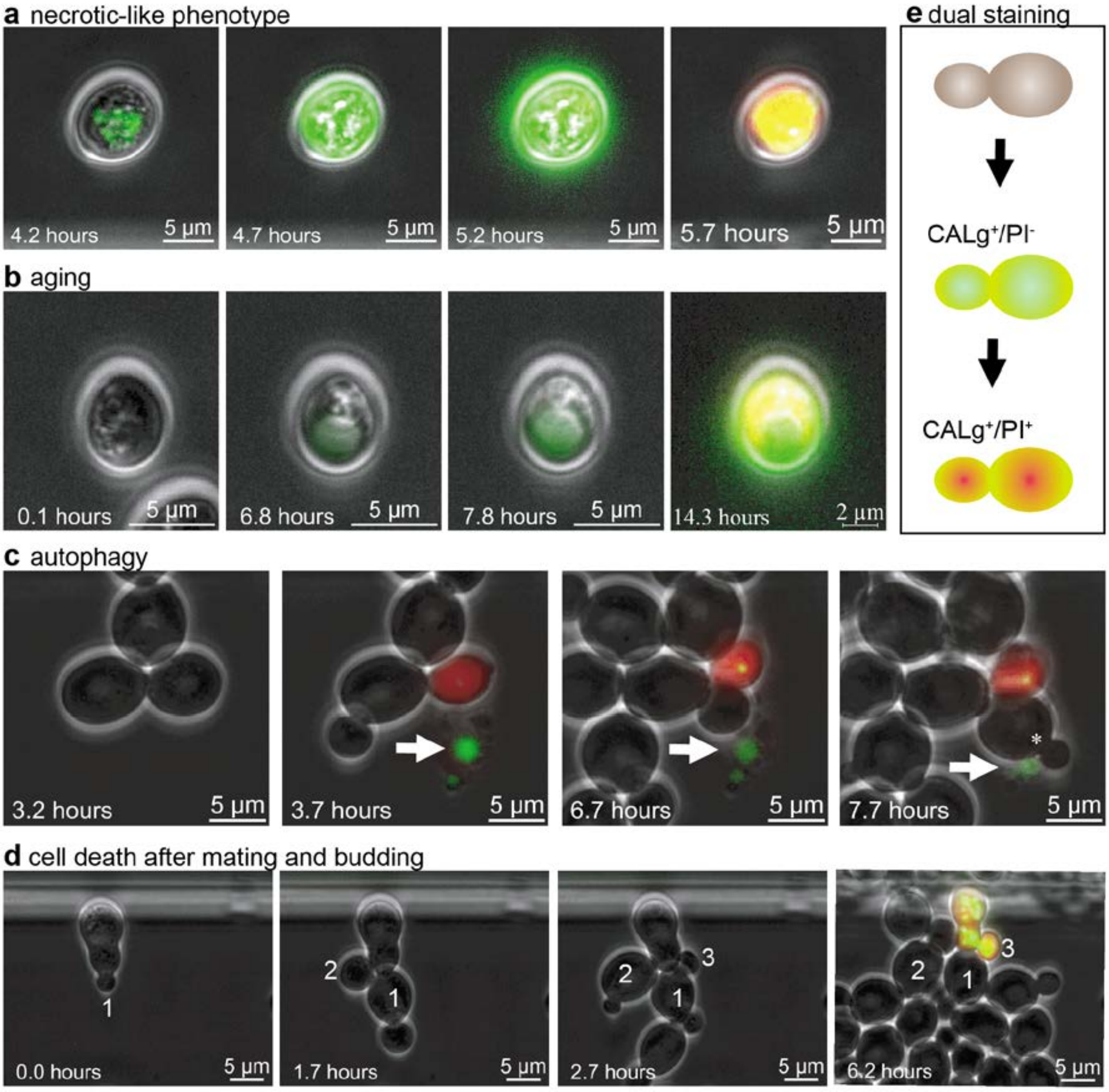

Figure S9 PI combined with calcein green to indicate apoptosis in yeast cells. (a) A yeast cell with a necrotic-like phenotype exhibited increased calcein green fluorescence (CALg$^+$) during the induction of apoptosis until the cell membrane ruptured, and calcein was not retained intracellularly (indicated by a light blue circle). PI fluorescence increased immediately after cell disintegration (PI$^+$). **(b)** Vacuole enlargement and an increase in vacuole pH characterized ageing in yeast, which was indicated by a local increase in calcein fluorescence. **(c)** A cell that formed two autophagy vesicles (indicated with a white arrow) labelled by calcein immediately prior to cell death (PI$^+$) is shown. The vesicles remained stable for hours before disappearing after cell contact with budding yeast (marked with *). **(d)** A budding zygote that generated two normally growing daughter cells (indicated with 1 and 2) is shown. After the third bud (3) formed, the yeast cell died, along with the bud. **(e)** The progression of PI/CgAM labelling is shown. The entire cell will be stained green if calcein efflux is inhibited. Partial calcein accumulation in vacuoles occurs in aged cells. Ageing cells accumulate calcein green in their enlarged vacuoles, presumably due to the loss of V-ATPase. Cells are initially calcein-positive (CALg$^+$) before acquiring PI fluorescence (PI$^+$).

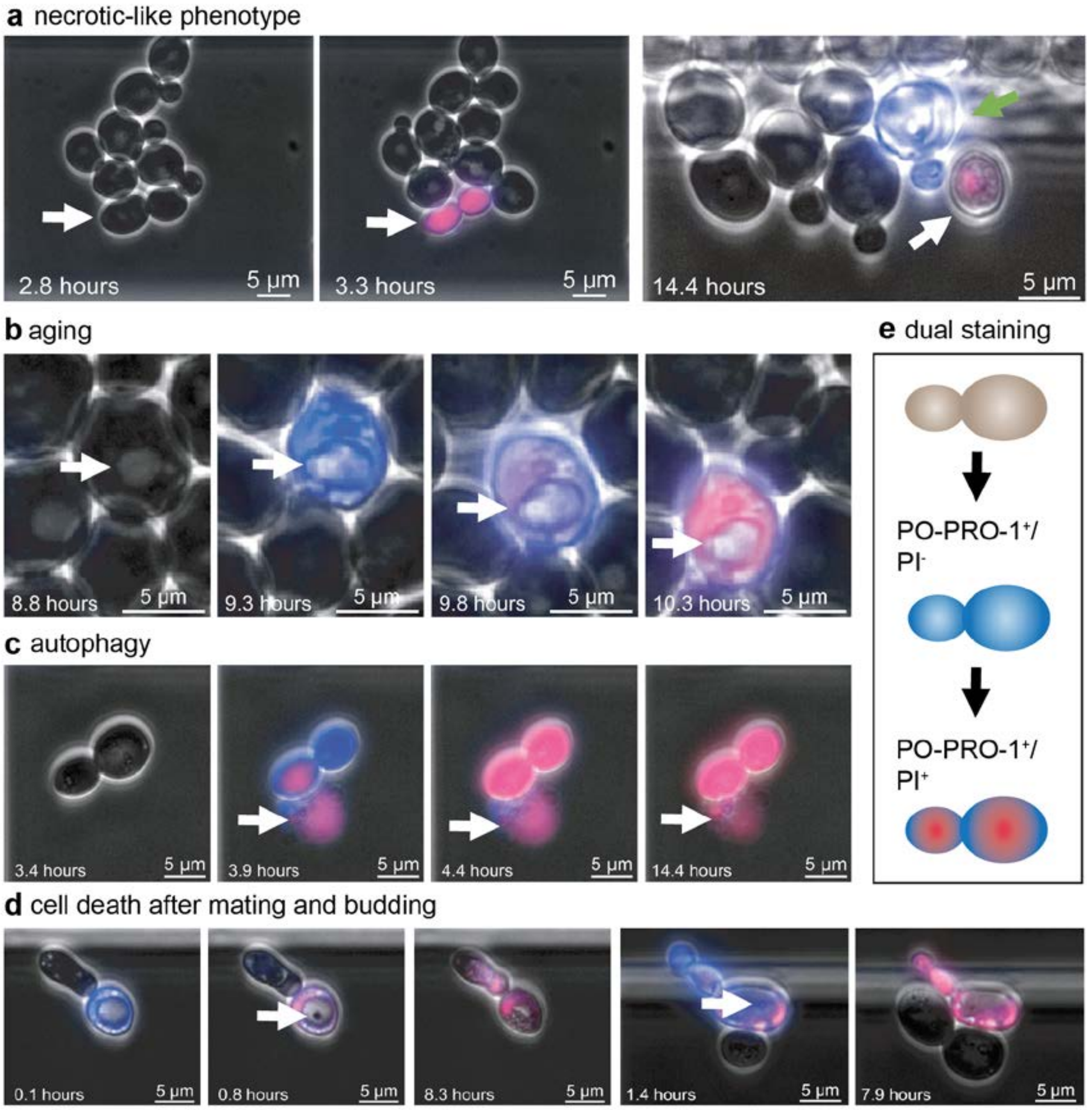

Figure S10 PI combined with PO-PRO-1 to indicate apoptosis in yeast cells. (a) Necrotic cells and late apoptotic necrotic-like cells both lose their membrane potential (PO-PRO-1^+) and cell integrity (PI^+) concurrently (white arrow). During apoptosis, cells induce DNA fragmentation, which reduces PI fluorescence (right image, white arrow), whereas early phase apoptotic cells lose their membrane potential (green arrow). **(b)** Although vacuoles are not stained by PI or PO-PRO-1, vacuole enlargement can be observed via phase-contrast imaging (white arrow). **(c)** A daughter cell that underwent macroautophagy produced an autophagosome containing DNA (white arrow) labelled by PI immediately prior to cell death (PI^+). The vesicles remained stable for hours prior to rupture. **(d)** Budding shmoos that mated with aged cells underwent apoptosis. The mating cells exhibited enlarged vacuoles and dense engulfment (white arrows). PI fluorescence transitioned from the mating cell to the associated shmoo. **(e)** The progression of PO-PRO-1/PI labelling is shown. Loss of membrane potential allows PO-PRO-1 to enter the cell and stain the entire cell intensely with blue fluorescence via DNA intercalation. Apoptotic cells are initially PO-PRO-1^+ before exhibiting PI fluorescence (PI^+).

Supplementary methods

Positive control of dynamic PI staining

After an initial growth phase under optimal growth conditions for *C. glutamicum, M. luteus, B. subtilis, E. coli, V. harveyi,* and *S. cerevisiae,* cells were continuously perfused with 50 mg/mL sodium cyanide and PO-PRO-1 to visualize the loss of membrane potential due to intoxication. After a substantial number of cells lost their membrane potential, dynamic PI staining was initiated. Signal-to-noise ratios were calculated as difference between the mean fluorescence and the mean background fluorescence divided by the standard deviation of the background fluorescence. The signal-to-background ratio was calculated as the ratio of the mean signal and the mean background fluorescence.

Additional endpoint staining

Additional endpoint staining of cells treated continuously with 50 µg/mL CHL was performed after 24 hours. Dynamic PI staining during antibiotic addition was combined with 10 µg/mL DAPI staining to visualize total DNA. Dynamic CvAM conversion due to CHL was endpoint-stained using 2 µg/mL Nile red (a membrane indicator). For Nile red staining, 10 µg/mL BSA was added to the perfusion medium for passivation. Both endpoint staining methods were performed with 0.15 % PFA for cell fixation.

Supplementary Videos

Video S1: Cell death and antibiotic tolerance of *Corynebacterium glutamicum* cells after the addition of antibiotics. Phenotypic analysis of *C. glutamicum* colonies during the continuous addition of antibiotics revealed a heterogeneous cell response. Cell death and extracellular DNA derived from lysed cells are indicated by red PI fluorescence. Lysed cells exhibited no fluorescence. Cells possessing residual metabolic activity indicated by an increase in blue CALv fluorescence remain in an antibiotic-tolerant state. A reference colony without the addition of antibiotics is shown for comparison.

Video S2: Programmed cell death of *Escherichia coli.* The initiation of single-cell death in *E. coli* BL21CodonPlus(DE3)-RIL cells producing a truncated lytic zeta toxin (PezT), an inactivated PezT or the antitoxin-toxin complex are indicated by red PI fluorescence. After

the induction of toxin expression with IPTG, cell survival (resistance) and the prior induction of non-lytic cell death were observed in a subpopulation that did not undergo complete cell lysis. Non-lytic single-cell death was also observed in *E. coli* cells expressing the inactivated toxin.

Video S3: Macroautophagy in *Saccharomyces cerevisiae*. Cell death (indicated by red PI fluorescence) after the induction of autophagy. PO-PRO-1 staining (blue fluorescence) indicates the loss of membrane potential and the presence of DNA fractions in the autophagosome. CALg (green fluorescence) accumulates in the autophagosome. CALg was generated from CgAM by intracellular esterases and was retained if no ATPase-mediated active transport was present.

Video S4: Ageing in *Saccharomyces cerevisiae*. Cell ageing of yeast is indicated by the enlargement and loss of function of vacuoles, which induces cell death. CALg (green fluorescence) accumulated in vacuoles that lost their vATPase activity. Cell death is indicated by PI (red fluorescence).

Video S5: Dying zygote. The sudden cell death (red PI fluorescence) of a bud can initiate a loss of function (green CALg fluorescence) and subsequent cell death.

Video S6: Shmoo-mediated mating of aged cells. Vacuole enlargement following nutrient stress likely appeared due to the ageing of yeast cells, resulting in cell death (red PI fluorescence). If shmoos mate with aged cells during programmed cell death, which initiates upon membrane potential loss (blue PO-PRO-1 fluorescence) followed by cell disintegration (red PI fluorescence), the mating shmoo dies following the cell death of the mating partner.

Video S7: Fluctuation in membrane potential followed by cell recovery. *Saccharomyces cerevisiae* cell recovery was observed following PO-PRO-1 (blue fluorescence) uptake. Cell budding was initiated, and the PO-PRO-1 that intercalated with the DNA was distributed between the mother and daughter cells.

Supplementary References

1. Grünberger, A. *et al.* Spatiotemporal microbial single-cell analysis using a high-throughput microfluidics cultivation platform. *J. Cytom. A* **87,** 1101–1115 (2015).

2. Westerwalbesloh, C. *et al.* Lab on a Chip Modeling and CFD simulation of nutrient distribution in picoliter bioreactors for bacterial growth studies on single-cell level. *Lab Chip* **15,** 4177–4186 (2015).

3. Unthan, S. *et al.* Beyond growth rate 0.6: What drives Corynebacterium glutamicum to higher growth rates in defined medium. *Biotechnol. Bioeng.* **111,** 359–71 (2014).

4. Grünberger, A. *et al.* Beyond Growth Rate 0.6: Corynebacterium glutamicum Cultivated in Highly Diluted Environments. *Biotechnol. Bioeng.* **110,** 18 (2013).

5. Grünberger, A. *et al.* Microfluidic Picoliter Bioreactor for Microbial Single Cell Analysis: Fabrication, System Setup and Operation. *J. Vis. Exp.* **82,** e50560 (2013).

6. Grünberger, A., Paczia, N., Probst, Christopher Schendzielorz, Georg Eggeling, L., Noack, S. & Wiechert, Wolfgang Kohlheyer, D. A disposable picolitre bioreactor for cultivation and investigation of industrially relevant bacteria on the single cell level. *Lab Chip* **12,** 9 (2012).

7. Stiefel, P., Schmidt-Emrich, S., Maniura-Weber, K. & Ren, Q. Critical aspects of using bacterial cell viability assays with the fluorophores SYTO9 and propidium iodide. *BMC Microbiol.* **15,** 1–9 (2015).

8. Lehtinen, J., Nuutila, J. & Lilius, E.-M. Green fluorescent protein-propidium iodide (GFP-PI) based assay for flow cytometric measurement of bacterial viability. *Cytom. Part A* **60,** 165–172 (2004).

9. Duineveld, P. C., Lilja, M., Johansson, T. & Inganäs, O. Diffusion of Solvent in PDMS Elastomer for Micromolding in Capillaries. *Langmuir* **18,** 9554–9559 (2002).

10. Warnes, S. L. & Keevil, C. W. Mechanism of Copper Surface Toxicity in Vancomycin-Resistant Enterococci following Wet or Dry Surface Contact **77,** 6049–6059 (2011).

11. Ullrich, S., Karrasch, B., Hoppe, H., Jeskulke, K. & Mehrens, M. Toxic effects on bacterial metabolism of the redox dye 5-cyano-2,3-ditolyl tetrazolium chloride. *Appl. Environ. Microbiol.* **62,** 4587–93 (1996).

12. Krämer, C. *et al.* Non-invasive microbial metabolic activity sensing at single cell level by perfusion of calcein acetoxymethyl ester. *PLoS One* **10,** e0141768 (2015).

13. Byrd, T. F. *et al.* The microfluidic multitrap nanophysiometer for hematologic cancer cell characterization reveals temporal sensitivity of the calcein-AM efflux assay. *Sci. Rep.* **4,** doi:10.1038/srep05117 (2014).

14. Lippuner, A. D., Julou, T. & Barral, Y. Budding yeast as a model organism to study the effects of age. *FEMS Microbiol. Rev.* **38,** 300–325 (2014).

15. Reggiori, F. & Klionsky, D. Autophagic processes in yeast: mechanism, machinery and regulation. *Genetics* **194,** 341–361 (2013).
